# Machine learning reveals limited predictive value of clinical factors for asthma exacerbations

**DOI:** 10.1038/s41598-025-19056-w

**Published:** 2025-10-08

**Authors:** Erik Duijvelaar, Jack S. Gisby, Hanneke Coumou, Jeroen Hoogland, Bart Hilvering, Anirban Sinha, Marijke Amelink, Els J. M. Weersink

**Affiliations:** 1 Department of Pulmonary Medicine, Amsterdam UMC Location Vrije Universiteit Amsterdam, 1081 HV Amsterdam, The Netherlands; 2 William Harvey Research Institute, Queen Mary University of London, London, UK; 3 Department of Epidemiology and Data Science, Amsterdam UMC Location Vrije Universiteit, De Boelelaan 1089A, Amsterdam, The Netherlands; 4 Immunology Discovery Research, AbbVie Cambridge Research Center, Cambridge, MA USA; 5 Department of Pulmonary Medicine, Spaarne Gasthuis, Boerhaavelaan 22, Haarlem, the Netherlands

**Keywords:** Asthma, Exacerbations, Predictive modelling, Machine learning, Risk stratification, Treatable traits, Respiratory tract diseases, Asthma

## Abstract

While predictors of asthma exacerbation risk are generally well established, predictors of exacerbation severity remain largely undefined. Identifying robust clinical predictors of exacerbation severity is essential to support tailored management strategies and optimize resource allocation. This study leverages machine learning to evaluate the predictive value of clinical factors for exacerbation severity in a real-world emergency department setting. A retrospective cohort study was performed using medical records of 367 adults (644 exacerbations) who presented to the Amsterdam UMC emergency department between 2013 and 2020. Five severity outcomes were investigated: hospital admission, ICU admission, length of stay, oxygenation efficiency (SpO₂/FiO₂), and National Early Warning Score (NEWS). Associations were assessed using linear mixed models (LMM), and predictive modelling employed a machine learning approach combining LMMs with fivefold cross-validated least absolute shrinkage and selection operator (LASSO) regression. Exacerbation severity was most consistently associated with lung function, the presence of a radiographic chest infiltrate, C-reactive protein levels, blood neutrophil count and theophylline maintenance use. No significant associations were found for blood eosinophil count, age, comorbidities, symptom duration, triggers, allergic sensitization, ethnicity or exacerbation history within the preceding 12 months. Internally validated prediction models for hospital and intensive care admission achieved areas under the curve of 0.632 and 0.695, respectively. The strongest predictors explained 18.8% of variability in NEWS, 15.2% in oxygenation efficiency, and 9.0% in length of hospital stay. In these prediction models, a radiographic chest infiltrate, followed by theophylline maintenance use and blood neutrophil count, were most frequently associated across the five severity outcomes. To conclude, lung function and markers of acute respiratory infection were most frequently associated with asthma exacerbation severity. However, clinical and demographic variables have only modest predictive value, highlighting the need to identify additional robust predictors.

**Supplementary Information:**

The online version contains supplementary material available at 10.1038/s41598-025-19056-w.

## Introduction

Asthma is a chronic inflammatory airway disease typically characterized by episodes of respiratory distress, known as exacerbations. Exacerbations comprise acute or subacute worsening in asthma symptoms (coughing, wheezing, dyspnoea and chest tightness) and lung function, attributable to increased airway constriction and/or inflammation^1^. They occur frequently in patients with severe or uncontrolled asthma and contribute to accelerated loss of lung function and a lower quality of life^2,3^.

In the last two decades, asthma management targeting disease-specific inflammatory pathways has been proven successful in improving lung function, tapering of systemic steroids and preventing subsequent exacerbations^4–9^. On the other hand, asthma exacerbations are typically managed based on symptom severity rather than by underlying inflammatory mechanisms^1,10,11^. Standard treatment of severe exacerbations mainly consists of the correction of hypoxemia with oxygen and reversal of airflow limitation with inhaled beta-2-agonists, inhaled corticosteroids and in severe cases systemic corticosteroids and intravenous magnesium sulphate^1,10,11^. Given the heterogeneous and complex inflammatory processes underlying asthma, there is a growing need to move from a generalized approach to a more phenotype-directed treatment of exacerbations to improve clinical outcomes^12^. Eosinophil-targeted therapy already showed promising results^13^. However, this transition necessitates more precise identification of risk factors and treatable traits associated with severity of exacerbations.

Known risk factors for the development of asthma exacerbations include previous exacerbations, smoking, female sex, inadequate asthma management, elevated blood or sputum eosinophil levels, increased fractional exhaled nitric oxide (FeNO) levels, and several pre-existing comorbidities such as obesity, chronic sinus disease, obstructive sleep apnoea and gastroesophageal reflux^14–19^. However, it remains unclear whether these same factors are associated with the severity of exacerbations or adverse clinical outcomes. Identification of risk factors or patients at risk for severe exacerbations could help prevent delayed escalation of care and enable more patient-tailored therapeutic interventions and. To optimise predictive performance while minimizing overfitting, machine learning models that incorporate cross-validation could effectively handle high-dimensional data and account for collinearity^20^. Therefore, the aim of this study is to identify clinical factors associated with exacerbation severity and clinical outcomes and to determine their predictive value using advanced s machine learning modelling.

## Methods

### Study population and design

Data from electronic health records were retrospectively collected for patients who presented to the emergency department at Amsterdam University Medical Center, location Academical Medical Center (AMC), between October 1, 2013, and March 1, 2020, with an asthma exacerbation. These patients were identified using asthma diagnosis treatment combinations, which are physician-made diagnoses that are generally followed by a sequence of medical activities and follows the International Statistical Classification of Diseases and Related Health Problems (ICD). Adult patients, 18 years or older at presentation, were considered eligible for study inclusion if the diagnosis of asthma was based on a history of confirmed variable expiratory airflow limitation and characteristic pulmonary symptoms, verified by at least one of the methods described by the latest report of the Global Initiative for Asthma^1^, and the exacerbation was characterized by acute or subacute worsening of asthma-related symptoms resulting in a physician reported diagnosis of asthma exacerbation or status asthmaticus. Once a patient was eligible for study inclusion by two independent physicians, chart data was pseudonymized. Ethical approval for study conduction was obtained from the ethical committee of the Amsterdam University Medical Centers (Amsterdam UMC), location Academic Medical Center (AMC). All methods were performed in accordance with relevant guidelines and regulations. Written informed consent was waived by the ethical committee. Instead, patients were informed about the study through a letter and were subsequently given the opportunity to object to inclusion by either email or returning a reply envelope.

### Study assessments

Study parameters include demographics, patient characteristics, pre-existent comorbid diseases, medication use, requirement for oxygen supplementation (eTable 2), and laboratory, microbiological and radiographic findings. The National Early Warning Score (NEWS) was calculated using the scoring system developed by the Royal College of Physicians^21^. Information on lung function was gathered using spirometry measurements outside of the exacerbation period. Peak expiratory flow data was collected from handheld peak-flow meters at presentation. The total dosage of inhalation corticosteroids was calculated as beclomethasone equivalent dosage (BED) using the conversion factors presented in supplementary eTable 3. A complete overview of all study parameters and their definitions is provided in supplementary eTable 1.

### Outcome measures

The severity of asthma exacerbations was determined by the ratio of peripheral oxygen saturation to the inspired fraction of oxygen (SpO2/FiO2), NEWS at emergency presentation, need for hospital admission, length of hospital stay and admission to the intensive care unit (ICU).

### Statistical analyses

All statistical analyses were performed using R, version 4.2.2 and RStudio version 2023.03.0 Cherry Blossom. Descriptive statistics were used to summarize patient and exacerbation characteristics. If a patient had multiple exacerbations during the study time period, the first visit was used to extract patient’s baseline characteristics. The baseline characteristics of patients stratified by either hospital admission or ICU admission status were compared using an unpaired T-test, the Mann–Whitney U test and the Chi-square test, for normally distributed numeric variables, non-normally distributed numeric variables and categorical variables respectively. Associations between patient and exacerbation characteristics and outcome measures were assessed using linear mixed models (LMMs), with random intercepts assigned to each patient to account for repeated measures. All other variables were included as fixed effects. LMMs were implemented using the lmer function from the lme4 package^22^. For the linear mixed models, the Benjamini–Hochberg procedure was used to adjust for multiplicity with a false discovery rate (FDR) set at 0.05.

To select the parameters with the highest predictive value, we modelled the data using Least Absolute Shrinkage and Selection Operator (LASSO) regression. This was done by using the glmmLasso function from the glmmLasso package^20^. This allowed for selection of a sequence of predictors with the highest independent predictive value. Random intercepts were given to each subject, all other study variables were included as fixed effects. Since missing data is not allowed for these prediction models, variables containing more than 15% missing values were excluded from LASSO regression analyses. For the variables CRP, duration of symptoms, body mass index (BMI), eosinophil, neutrophil and leukocyte blood count, missing values were imputed with the mean or median depending on the distribution. For categorical variables, an additional category “unknown” was added. The LASSO models were first fit during fivefold cross validation on a train set of patients, composed of 80% of randomly selected patients. Then, these models were tested on the remaining 20% of patients, comprising the hold-out validation cohort. The final LASSO model was then fit on the full dataset. Finally, the selected variables were included in a final LMM model. For all statistical tests, a p-value of < 0.05 was considered statistically significant.

## Results

### Cohort characteristics

**Fig. 1:**
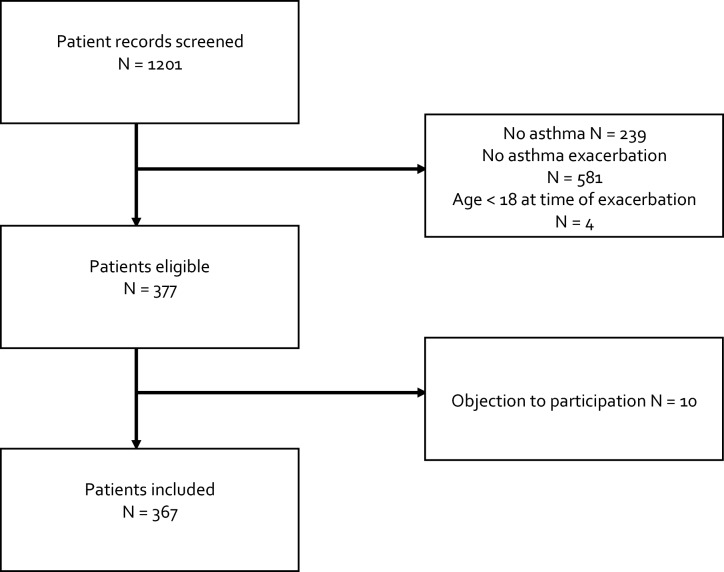
Study flow diagram. Flow diagram of patient screening and inclusion.

The medical records of 1201 patients were screened, of which 824 patients were excluded due to not meeting the inclusion criteria (Fig. 1). From the 377 eligible patients, 10 patients were further excluded because they objected to their data being used for this study. A total of 367 patients were included, which had presented to the emergency department with a total of 644 exacerbations. The proportion of missing data for each included variable is provided in eTable 4.

The cohort was predominantly female (60%), with a mean age of 45.1 ± 17.8 years (Table 1). The majority of patients were Caucasian (55%), 47% had a history of smoking or were current smokers, and 68% had an allergy to aeroallergens. Additionally, 52 patients (14%) were concomitantly diagnosed with COPD. While most patients presented once during the study period, 117 (32%) experienced more than one exacerbation (Table 1). Exacerbations occurred most frequently during winter (31.1%; χ^2^ = 11.77, p = 0.0003, Table 2). The median duration of symptoms before patients visited the emergency department was 4 days [IQR 2, 14]. Maintenance inhalation corticosteroids (ICS) were used before 482 (74.8%) of exacerbations, with a median dosage of 1000 µg BED [IQR 0, 2000]. During almost half (47.2%) of the exacerbations, patients reported flu-like symptoms. In 135 exacerbations, a nasal and/or throat swab was performed, which tested positive for a respiratory virus in 84 exacerbations (62.2%). A bacterial infection was cultured in 23 out of the 141 (16.3%) sputum samples. A radiographic infiltrate was observed in 65 (11.3%) exacerbations. Median blood eosinophil count was 0.15 [0.04, 0.39] × 10^9/L and median CRP level was 5.9 [2.2, 16.5] mg/L. From all 644 exacerbations, 279 (43.3%) required hospital admission, 29 exacerbations (4.5%) led to ICU admission and 7 (1.1%) patients required invasive ventilation (Table 2 and eTable 5). None of the patients died due to the asthma exacerbation or its complications.

**Table 1:** Patient characteristics.

Patient characteristics		N = 367
Age (years)		45.1 ± 17.8
Female Sex		220 (60%)
Weight (kg) N = 266		82.8 ± 18.0
Height (cm) N = 279		168 ± 10
Body Mass Index (kg/m2) N = 264		29.4 ± 6.7
Ethnicity N = 339		
	Caucasian	185 (55%)
	Black	52 (15%)
	Asian or pacific islander	23 (7%)
	Hispanic	9 (3%)
	Mixed and other	69 (19%)
Smoking N = 352		
	Never smoked	188 (53%)
	Past smoker	80 (23%)
	Current smoker	84 (24%)
Comorbidities		
	GERD	56 (15%)
	COPD	52 (14%)
	Rhinitis	122 (33%)
	CRSwNP	37 (10%)
	CRSsNP	35 (10%)
	bronchiectasis	21 (6%)
	OSAS	34 (9%)
	Urticaria	18 (5%)
Allergies		
	Aeroallergens N = 363	246 (68%)
	Aspergillus Fumigatus N = 148	36 (24%)
Multiple (≥ 2) exacerbations		117 (32%)

The total number of patients used to describe proportions is 367, unless stated otherwise. Data is presented as proportion (percentage) or mean ± SD. COPD = Chronic Obstructive Pulmonary Disease, CRSsNP = Chronic rhinosinusitis without nasal polyps, CRSwNP = Chronic rhinosinusitis with nasal polyps, GERD = Gastroesophageal Reflux Disease, OSAS = Obstructive Sleep Apnea Syndrome.

**Table 2:** Exacerbation characteristics at emergency presentation.

Characteristic	All exacerbations N = 644
**Exacerbation history**	
Time since previous emergency department visit due to an asthma exacerbation (days) N = 227	84 [32, 208]
Time since previous hospital admission (days) N = 146	102 [37, 209]
Duration of symptoms (days) N = 634	4 [2,14]
**Season**	
Spring	145 (22.5%)
Summer	149 (23.1%)
Autumn	150 (23.3%)
Winter	200 (31.1%)
**Daily maintenance medication use**	
Inhalation Corticosteroid	486 (75.5%)
Inhalation Corticosteroid dosage (in mcg BED)	1000 [0, 2000]
Long-Acting Muscarinic Antagonist	176 (27.3%)
Leukotriene Receptor Antagonists	98 (15.2%)
Theophylline	14 (2.2%)
Corticosteroid maintenance therapy	159 (24.7%)
Inhalation Corticosteroid category	
0–200 mcg BED	162 (25.2%)
201–500 mcg BED	53 (8.2%)
501–1000 mcg BED	119 (18.5%)
> 1000 mcg BED	310 (48.1%)
Use of any biological	64 (9.9%)
Mepolizumab	35 (5.4%)
Omalizumab	22 (3.4%)
Reslizumab	6 (0.9%)
Benralizumab	0 (0%)
Dupilumab	1 (0.2%)
**Blood markers**	
Eosinophil (× 10^9/L) N = 558	0.15 [0.04, 0.39]
Neutrophil (× 10^9/L) N = 545	7.02 ± 3.53
Leukocyte (× 10^9/L) N = 585	10.2 ± 3.9
CRP (mg/L) N = 574	5.9 [2.2, 16.5]
**Patient reported triggers**	
Flu-like symptoms	339 (52.6)
Allergen exposure	51 (7.9%)
Non-specific trigger	61 (9.5%)
Occupational exposure	2 (0.3%)
**Lung function at presentation**	
Peak expiratory flow (L/min) N = 355	241 ± 127
Peak expiratory flow % of predicted N = 355	45.0 ± 21.1
**Radiologic findings**	
Infiltrate on X-ray or computed tomography-scan N = 576	65 (11.3%)
**Viral and bacterial infections**	
Any viral infection N = 135	84 (62.2%)
Any bacterial infection N = 140	23 (16.3%)
**Arterial blood gas and oxygenation**	
pH in arterial blood N = 379	7.46 ± 0.08
PaCO2 in arterial blood (kPa) N = 379	4.62 ± 1.44
PaO2 in arterial blood (kPa) N = 379	11.8 ± 4.7
Oxygen saturation (%) in arterial blood N = 379	94.2 ± 3.2
SpO2, % N = 638	96 [93, 98]
Fraction of inspired oxygen (FiO2, %)	20 [20]
SpO2/FiO2 N = 638	475 [460, 490]
PaO2/FiO2 (mmHg) N = 265	399 ± (141)
**Clinical outcome and disease severity**	
National Early Warning Score (NEWS) N = 637	3 [1, 5]
Admission	279 (43.3%)
Length of hospital stay in admitted patients (days) N = 264	3 [2, 6]
Intensive care admission	29 (4.5%)
Intubation	7 (1.1%)
Mortality	0 (0%)

The total number of exacerbations used for analysis was 644, unless stated otherwise. Data is presented as proportion (percentage), mean ± SD or median [IQR]. BED = Beclomethasone Equivalent Dose, CRP = C-reactive protein, FiO2 = Fraction of inspired oxygen, PaCO2 = Partial pressure of carbon dioxide, PaO2 = Partial pressure of Oxygen, SpO2 = Saturation of Peripheral Oxygen.

### Characteristics associated with hospital admission

First, we identified the characteristics of exacerbations that required hospital admission. A summary is provided in Table 3, a complete overview is provided in eTable 6. Patients who were admitted had significantly more emergency department visits (p = 0.007) and hospital admissions (p = 0.001) in the 12 months prior to presentation. There were no significant differences in differential leukocyte counts, nor in CRP levels between the two groups. The exacerbations that required admission were characterized by more severe obstruction, as indicated by the lower percentage of predicted peak expiratory flow as assessed by a peak flow meter (p = 0.042). Their lung function, assessed outside of the exacerbation period, showed a significantly lower percentage of predicted FEV1 (p = 0.025) and FEV1/FVC (p = 0.001). A pulmonary infiltrate, detected either on a chest X-ray or CT-scan, was more often present in exacerbations that required admission (p = 0.001). However, admissions were not more often associated with viral or bacterial infections.

**Table 3:** Stratification of patients by admission status.

	Not admitted	Admitted	P-value
	N = 365	N = 279	
**Patient characteristics**			
Age (years)	44.0 ± 17.5	45.4 ± 17.2	0.298
Body Mass index (kg/m2), N = 519	29.1 ± 6.0	29.8 ± 6.5	0.268
Female	221 (60.5%)	190 (68.1%)	0.058
Smoking status, N = 626			0.371
Never smoked	197 (56.1%)	146 (53.0%)	
Past smoker	86 (24.5%)	81 (29.5%)	
Current smoker	68 (19.4%)	48 (17.5%)	
**Allergy**			
Aeroallergens	235 (68.1%)	187 (69.5%)	0.777
**Exacerbation characteristics**			
Number of asthma exacerbations 12 months prior	0.71 ± 1.46	1.03 ± 1.83	**0.014**
Number of emergency department visits 12 months prior	0.7 ± 1.3	1.0 ± 1.8	**0.007**
Number of hospital admissions 12 months prior	0.3 ± 0.8	0.6 ± 1.2	**0.001**
Time since last hospital admission (days), N = 146	118 [73, 245]	92 [31, 191]	**0.017**
**Medication use**			
Oral steroid maintenance	70 (19.2%)	89 (31.9%)	** < 0.001**
Long-Acting Muscarinic Antagonist	79 (21.6%)	97 (34.8%)	** < 0.001**
**Blood counts**			
CRP (mg/l), N = 574	5.40 [1.85, 13.75]	6.50 [2.75, 18.95]	0.075
Eosinophil (× 10^9/L), N = 558	0.17 [0.07, 0.39]	0.14 [0.02, 0.39]	0.106
Neutrophil (× 10^9/L), N = 545	6.74 (3.28)	7.32 (3.76)	0.056
Leukocyte (× 10^9/L), N = 585	9.94 (3.60)	10.10 [7.43, 12.65]	0.100
**Lung function**			
Peak Expiratory Flow (% of predicted), N = 140	48.2 ± 19.2	40.9 ± 22.9	**0.042**
FEV1% of predicted, N = 526	78.6 ± 20.4	74.5 ± 21.4	**0.025**
FEV1/FVC index, N = 528	0.70 ± 0.13	0.66 ± 0.13	**0.002**
**Radiologic findings**			
Infiltrate on X-ray or computed tomography-scan (%) N = 576	22 (7.1%)	43 (16.1%)	**0.001**
**Infection**			
Any virus, N = 135	30 (61.2%)	54 (62.8%)	1.000
Parainfluenza, N = 135	4 (3.0%)	0 (0.0%)	**0.031**
Any bacteria, N = 141	9 (6.4)	14 (9.9)	0.993
**Clinical outcomes and disease severity**			
SpO2 (pulse oximetry), N = 638	96.0 [94.0, 98.0]	94.0 [92.0, 97.0]	** < 0.001**
pH, N = 379	7.45 [7.43, 7.49]	7.45 [7.42, 7.49]	0.434
PaO2 (kPa), N = 379	10.8 [9.4, 12.5]	9.9 [8.7, 13.4]	0.124
PaCO2 (kPa), N = 379	4.6 [4.1, 5.0]	4.6 [4.1, 5.2]	0.211
Oxygen saturation (%), N = 379	95.0 [93.4, 96.2]	94.4 [91.8, 96.8]	0.410
Hypercapnia, N = 379	1 (0.6)	14 (6.3)	**0.011**
FiO2, %	20.2 (1.1)	23.1 (9.1)	** < 0.001**
National Early Warning Score (NEWS), N = 622	2 [1, 4]	4 [2, 6]	** < 0.001**
SpO2/FiO2, N = 638	476.47 (25.72)	434.07 (82.28)	** < 0.001**

The total number of exacerbations used for analysis was N = 644, unless stated otherwise. Data is presented as proportion (percentage), mean ± SD or median [IQR]. FEV1 = Forced Expiratory Volume in 1 s, FVC = Forced Vital Capacity, PaO2 = Partial pressure of Oxygen, SpO2 = Saturation of Peripheral Oxygen.

### Characteristics associated with intensive care admission

Second, we examined the characteristics of exacerbations that required admission to the ICU (eTable 7), the variables that were statistically significant are provided in Table 4. Exacerbations that led to ICU admission were characterized by higher blood neutrophil (p < 0.001) and leukocyte counts (p = 0.004). These patients experienced a shorter duration of symptoms before they presented to the emergency department (p < 0.001). A rhinovirus infection occurred more often in patients who were admitted to the ICU (20% versus 60%, p = 0.002), the incidence of bacterial infections was not different. ICU admissions were characterized by lower SpO2 (p = 0.001), lower pH in arterial blood gas (p = 0.001), higher FiO2 (p < 0.001), higher PaCO2 (p = 0.007) and higher NEWS (p < 0.001; Table 4 and eTable 7).

**Table 4:** Stratification of patients by admission to the intensive care unit.

	Not admitted to ICU N = 615	Admitted to ICU N = 29	p-value
**Medication**			
Theophylline	9 (1.5%)	5 (17.2%)	** < 0.001**
**Exacerbation characteristics**			
Duration of symptoms (days)	4.00 [2.00, 14.00]	1.00 [0.00, 3.00]	** < 0.001**
**Season (%)**			**0.007**
Autumn	136 (22.1%)	14 (48.3%)	
Spring	143 (23.3%)	2 (6.9%)	
Summer	144 (23.4%)	5 (17.2%)	
Winter	192 (31.2%)	8 (27.6%)	
**Exacerbationtrigger**			
Non-specific trigger	54 (8.8%)	7 (24.1%)	**0.015**
Rhinovirus infections	24 (20.0%)	9 (60.0%)	**0.002**
**Blood counts**			
Neutrophil (× 10^9/L), N = 545	6.20 [4.47, 8.62]	9.94 [6.52, 12.47]	** < 0.001**
Leukocyte (× 10^9/L), N = 585	9.60 [7.50, 12.03]	12.30 [9.20, 16.00]	**0.004**
**Clinical outcomes and disease severity**			
SpO2, N = 638	96.00 [93.00, 98.00]	92.00 [89.00, 97.00]	**0.001**
FiO2 (%)	20.00 [20.00, 20.00]	20.00 [20.00, 32.00]	** < 0.001**
SpO2/FiO2, N = 638	462.97 ± 51.12	360.50 ± 132.18	** < 0.001**
pH N = 379	7.45 [7.43, 7.49]	7.41 [7.36, 7.47]	**0.001**
Hypercapnia, N = 379	8 (2.3%)	7 (24.1%)	** < 0.001**
PaCO2 (kPa), N = 379	4.60 [4.10, 5.00]	5.10 [4.40, 6.00]	**0.007**
NEWS, N = 622	3.00 [1.00, 5.00]	7.00 [4.75, 8.25]	** < 0.001**

The total number of exacerbations used for analysis was N = 644, unless stated otherwise. Data is presented as proportion (percentage), mean ± SD or median [IQR]. FiO2 = Fraction of inspired oxygen, ICU = Intensive Care Unit, NEWS = National Early Warning Score, PaCO2 = Partial pressure of carbon dioxide, SpO2 = Saturation of Peripheral Oxygen.

### Associations with five exacerbation severity outcomes

Third, we examined the univariate associations between patient and exacerbation characteristics and five key outcomes indicative of exacerbation severity: hospital admission, length of hospital stay, admission to the intensive care unit (ICU), NEWS score, and the SpO2/FiO2 ratio (Table 5). In contrast with the previous regression models, these models accounted for within-patient variability. Due to the wide range of viral and bacterial infections, only those with significant associations with one or more outcomes are reported. Comorbidities, seasonal categories, biological factors, ethnicity, and exacerbation history—including emergency presentations, hospitalizations, and steroid courses within the prior 12 months—are not presented here as none demonstrated significant associations. Full details are provided in eTable 8.

**Table 5:** Associations between study characteristics and disease severity and clinical outcomes.

Parameter	Admission		Intensive care admission		Length of hospital stay		Oxygenation efficiency (SpO2/FiO2)		NEWS	
	Estimate (SD)	Adjusted p-value	Estimate (SD)	Adjusted p-value	Estimate (SD)	Adjusted p-value	Estimate (SD)	Adjusted p-value	Estimate (SD)	Adjusted p-value
**Demographic characteristics**										
Age	0.002 (0.001)	0.369	−0.001 (0.001)	0.580	0.02 (0.01)	0.137	−0.18 (0.17)	0.830	0.01 (0.01)	0.305
Smoking status	NA	0.769	NA	0.950	NA	0.351	NA	0.019	NA	0.292
Past smoker	0.069 (0.054)	NA	0.008 (0.023)	NA	0.59 (0.38)	NA	−12.14 (7.05)	NA	0.51 (0.28)	NA
Current smoker	0.017 (0.058)	NA	0.009 (0.024)	NA	−0.36 (0.41)	NA	−27.36 (7.45)	NA	0.54 (0.29)	NA
Body mass index (kg/m^**2**^**)**	0.004 (0.004)	0.690	0.003 (0.002)	0.580	0.09 (0.03)	0.022	0.10 (0.52)	0.987	0.02 (0.02)	0.670
Ethnicity	NA	0.862	NA	0.817	NA	0.801	NA	0.987	NA	0.739
Sex (female)	0.059 (0.046)	0.588	−0.002 (0.020)	0.950	0.40 (0.33)	0.548	19.05 (5.98)	0.020	−0.21 (0.24)	0.670
Season	NA	0.864	NA	0.137	NA	0.827	NA	0.987	NA	0.957
**Allergy**										
Allergic sensitization to aeroallergens	−0.005 (0.048)	0.958	−0.018 (0.020)	0.817	−0.78 (0.34)	0.137	6.03 (6.37)	0.917	−0.25 (0.25)	0.638
Allergic sensitization to Aspergillus	−0.140 (0.077)	0.329	−0.067 (0.043)	0.580	−0.77 (0.62)	0.548	2.10 (10.02)	0.987	0.28 (0.41)	0.720
**Radiologic findings**										
Infiltrate on X-ray or CT -scan	0.235 (0.066)	0.024	0.030 (0.029)	0.756	1.51 (0.45)	0.022	−17.31 (8.38)	0.212	1.50 (0.32)	0.000
**Exacerbation characteristics**										
Duration of symptoms (days)	0.000 (0.001)	0.910	0.000 (0.000)	0.620	0.00 (0.00)	0.727	0.13 (0.08)	0.376	−0.01 (0.00)	0.052
Flu-like symptoms	0.022 (0.039)	0.827	−0.002 (0.016)	0.950	0.13 (0.26)	0.801	−2.11 (4.70)	0.987	0.42 (0.19)	0.134
Non-specific trigger exposure	−0.003 (0.067)	0.969	0.067 (0.028)	0.137	−0.19 (0.44)	0.827	13.07 (8.02)	0.403	−0.45 (0.32)	0.451
Allergen exposure	−0.077 (0.072)	0.639	−0.020 (0.030)	0.819	−0.79 (0.49)	0.351	1.88 (8.78)	0.987	−0.55 (0.35)	0.346
**Lung function at presentation**										
Peak flow % of predicted	−0.004 (0.002)	0.216	−0.001 (0.001)	0.756	−0.01 (0.01)	0.591	0.31 (0.23)	0.614	−0.03 (0.01)	0.001
**Lung function prior presentation**										
FEV1% of predicted	−0.003 (0.001)	0.183	−0.001 (0.001)	0.756	−0.02 (0.01)	0.111	0.55 (0.15)	0.007	−0.02 (0.01)	0.003
FVC % of predicted	0.000 (0.001)	0.910	0.000 (0.001)	0.819	−0.01 (0.01)	0.351	0.31 (0.17)	0.307	−0.02 (0.01)	0.076
FEV1/FVC	−0.503 (0.181)	0.086	−0.069 (0.083)	0.819	−2.20 (1.28)	0.351	103.50 (23.47)	0.001	−3.63 (0.93)	0.001
**Use of maintenance therapy**										
ICS dosage in mcg BED × 1000	0.000 (0.000)	0.933	0.000 (0.000)	0.756	0.00 (0.00)	0.866	0.01 (0.00)	0.022	0.00 (0.00)	0.305
Using theophylline	0.220 (0.143)	0.460	0.275 (0.059)	0.000	1.05 (0.99)	0.637	−41.46 (18.88)	0.188	2.76 (0.74)	0.002
Using inhaled LAMA	0.124 (0.050)	0.138	−0.027 (0.022)	0.714	1.07 (0.35)	0.040	2.07 (6.54)	0.987	0.48 (0.26)	0.240
Using LTRA	−0.004 (0.061)	0.967	−0.011 (0.026)	0.853	−0.08 (0.43)	0.911	11.36 (7.88)	0.549	−0.82 (0.31)	0.052
Using oral steroids	0.142 (0.051)	0.086	0.007 (0.022)	0.906	0.71 (0.36)	0.251	0.24 (6.64)	0.987	0.19 (0.26)	0.720
Using a biologica	−0.075 (0.070)	0.639	−0.041 (0.029)	0.620	−0.25 (0.47)	0.801	0.35 (8.66)	0.987	0.06 (0.35)	0.957
**Blood investigations**										
Leukocyte blood count	0.010 (0.005)	0.276	0.009 (0.002)	0.002	0.06 (0.04)	0.384	−1.71 (0.66)	0.078	0.12 (0.03)	0.000
Eosinophil blood count	0.035 (0.059)	0.827	0.003 (0.026)	0.950	−0.02 (0.42)	0.973	−6.44 (7.71)	0.987	−0.28 (0.30)	0.670
Neutrophil blood count	0.013 (0.006)	0.216	0.013 (0.003)	0.000	0.09 (0.04)	0.182	−2.63 (0.75)	0.009	0.15 (0.03)	0.000
C-reactive protein (CRP)	0.002 (0.001)	0.030	0.000 (0.000)	0.819	0.00 (0.00)	0.717	−0.03 (0.08)	0.987	0.01 (0.00)	0.001
**Infections**										
Any viral infection	0.019 (0.086)	0.910	0.021 (0.054)	0.855	−0.24 (0.83)	0.866	−5.03 (11.84)	0.987	0.28 (0.49)	0.752
Rhinovirus infection	0.079 (0.097)	0.769	0.191 (0.060)	0.023	0.11 (0.96)	0.951	−6.80 (13.28)	0.987	0.44 (0.55)	0.720
Enterovirus infection	0.368 (0.344)	0.639	0.645 (0.185)	0.015	2.04 (3.26)	0.801	15.02 (45.32)	0.987	−2.56 (1.94)	0.466
Any bacterial infection	−0.061 (0.108)	0.827	−0.036 (0.073)	0.819	0.99 (0.96)	0.650	3.11 (17.63)	0.987	−0.69 (0.61)	0.570
Pseudomonas spp infection	0.359 (0.319)	0.639	−0.108 (0.227)	0.819	9.46 (2.81)	0.022	−25.40 (52.97)	0.987	−0.28 (1.85)	0.957

BED = Beclomethasone Equivalent Dose, CT = Computed Tomography, FiO2 = Fraction of inspired oxygen, FEV1 = Forced Expiratory Volume in 1 s, FVC = Forced Vital Capacity, ICS = Inhalation Corticosteroid, LAMA = Long-Acting Muscarinic Antagonist, LTRA = Leukotriene Receptor Antagonists, NEWS = National Early Warning Score, SpO2 = Saturation of Peripheral Oxygen.

### Predictive modelling

The presence of an infiltrate on a chest X-ray or CT-scan was associated with higher NEWS scores, more frequent hospital admissions, and longer hospital stays (Table 5). Worse pulmonary function, specifically a lower FEV1 and FEV1/FVC, was associated with higher NEWS and worse oxygenation efficiency. CRP levels were positively associated with NEWS and hospital admissions. Blood neutrophil count and total leukocyte count were associated with higher NEWS and ICU admission although blood eosinophil counts showed no significant association with any outcomes. Overall, the presence of viral or bacterial infections was not associated with the studied outcomes. However, rhinovirus and enterovirus infections were specifically linked to ICU admission, and pseudomonas infection or colonization was associated with prolonged hospital stays (Table 5).

Lastly, we aimed to identify parameters that could predict our clinical outcomes of interest. To achieve this, we applied LASSO regression to select the most important predictors from the linear mixed models. A detailed overview of the variables selected by each model, along with their test characteristics, is provided in eTables S9–S13. Table 6 contains the variables that achieved statistical significance. The presence of an infiltrate on chest X-ray or CT scan emerged as an independent and significant predictor for all outcomes except ICU admission, for which a trend with p = 0.050 was observed (eTable 10). Blood neutrophil count was selected for the prediction of ICU admission and oxygenation efficiency, whilst eosinophil count only emerged as a significant predictor for oxygenation efficiency. CRP levels were a significant predictor for hospital admission only. These predictors were largely consistent in a cohort of patients without comorbid COPD (eTable 14). For hospital admission and length of hospital stay, all previously identified predictors remained statistically significant. For ICU admission, NEWS, and oxygenation efficiency, one predictor in each model (non-specific trigger, duration of symptoms, and neutrophil count, respectively) approached but did not meet the threshold for statistical significance (p-values: 0.06, 0.052, and 0.054, respectively).

**Table 6:** P-values of the variables selected in the LASSO models to predict the severity and outcomes of asthma exacerbations.

Variable	hospital admission	ICU admission	NEWS	Oxygenation efficiency (SpO2/FiO2)	Length of hospital stay
Infiltrate on X-ray or CT-scan	0,000	ns	0,000	0,002	0,021
C-reactive protein (CRP)	0,012	ns	ns	ns	ns
Using oral corticosteroids	0,028	ns	ns	ns	ns
Using theophylline	ns	0,000	0,000	0,005	ns
Neutrophil blood count	ns	0,001	ns	0,030	ns
Season	ns	0,018	ns	ns	ns
Non-specific trigger exposure	ns	0,022	ns	ns	ns
Using leukotriene receptor antagonists	ns	ns	0,000	ns	ns
Duration of symptoms	ns	ns	0,002	ns	ns
Eosinophil blood count	ns	ns	ns	0,035	ns
Smoking status	ns	ns	ns	0,046	ns
Dosage of inhalation corticosteroids	ns	ns	ns	ns	0,039

CT = Computed Tomography, FiO2 = Fraction of inspired oxygen, ICU = Intensive Care Unit, NEWS = National Early Warning Score, ns = non-significant, SpO2 = Saturation of Peripheral Oxygen.

**Fig. 2:**
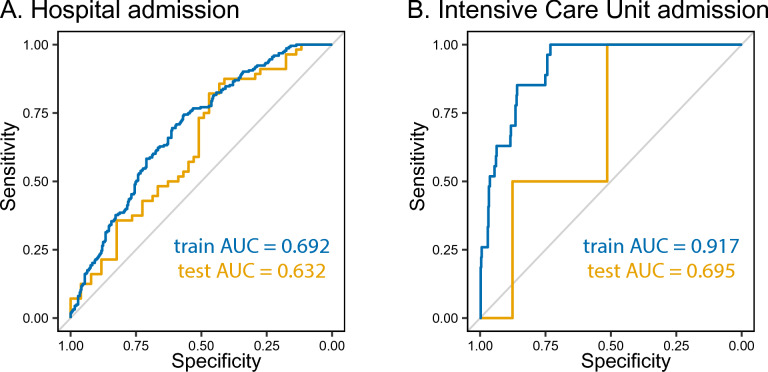
Performance of the train (blue) and test (orange) prediction models. Receiver operating curves showing the performance of the prediction models for hospital admission (**A**) and intensive care unit admission (**B**). AUC = Area under the curve.

For the two binary outcomes, hospital admission and ICU admission, receiver operating characteristic (ROC) curves were generated (Fig. 2). In a train cohort comprising 80% of randomly selected patients, the model yielded an area under the curve (AUC) of 0.692 for predicting hospital admission (Fig. 2A). On the hold-out validation cohort, the model achieved an AUC of 0.632. Although the AUC for predicting ICU admission was higher at 0.917 in the training cohort, the performance on the test cohort was substantially lower with an AUC of 0.695 (Fig. 2B). The proportion of variance explained by the selected variables in the test cohort, the fixed effects (R^2^m), was 18.8% for NEWS, 15.2% for oxygenation efficiency and 9.0% for length of hospital stay. The proportion of variance explained by both fixed and random (patient-specific variability) effects (R^2^c) was 36.3% for NEWS, 39.5% for oxygenation efficiency and 36.5% for length of hospital stay.

## Discussion

This retrospective study of adults presenting with asthma exacerbations to an emergency department of a large university hospital aimed to identify clinical predictors of exacerbation severity. While numerous studies have explored risk factors for developing subsequent exacerbations, research specifically focused on predictors of exacerbation severity remains limited^23^. Understanding these severity predictors is essential to enhance tailored clinical decision-making and optimize the allocation of healthcare resources. We determined the strongest predictors and their predictive value using cross-validated machine learning models applied to high-dimensional, real-world data. Our findings show that impaired baseline lung function and markers of acute infection-specifically, radiographic infiltrate, elevated CRP, and increased blood neutrophils-were most frequently associated with severe exacerbations. In contrast, factors such as blood eosinophil count, age, comorbidities, symptom duration, and exacerbation history were not significant predictors. Notably, our predictive models demonstrated only moderate accuracy. These findings highlight the complex interplay of factors influencing asthma exacerbation severity and the need to identify additional (bio)markers and patient-specific traits to improve risk stratification.

In our study, exacerbations requiring hospital admission frequently occurred in patients with a history of prior exacerbations, emergency department visits, or hospitalizations within the preceding 12 months. These findings align with previous studies identifying prior exacerbations as one of the strongest risk factors for recurrent exacerbations, often referred to as the ‘frequent exacerbator phenotype’^14–16,24,25^. However, in our study, these features were not associated with more severe exacerbations or worse clinical outcomes. These findings suggest that prior exacerbations, emergency visits, and hospitalizations are not independent risk factors for exacerbation severity, but instead reflect a distinct phenotype of patients prone to recurrent exacerbations or poor asthma control^26^.

Blood eosinophil count at hospital presentation was not independently associated with exacerbation severity in our study. Although it was selected as a predictor of oxygenation efficiency in the multivariate LASSO regression model, its very high p-value in the univariate mixed model indicates that eosinophil count is not an independent predictor. Moreover, the mean SpO₂/FiO₂ in exacerbations where eosinophil count was missing, and therefore imputed using the median, was significantly higher than in those with available data (455 vs. 481, p < 0.001). Imputation of these missing values with the median may have introduced bias, potentially leading to inclusion of eosinophil count in the LASSO models. Eosinophilic inflammation, with blood eosinophilia as a surrogate marker, is a well-established risk factor for future severe exacerbations and hospitalizations^27^, and even for the severity of concurrent exacerbations^28^. While this could be due to the high proportion of patients using inhaled corticosteroids (75.5%) or oral maintenance steroids (24.7%), our findings suggests that type 2 (T2) inflammation may not play a dominant role during exacerbations. This discrepancy may reflect the evolving understanding of asthma phenotypes, where neutrophilic and non–type 2 inflammatory pathways increasingly appear to drive severe exacerbations. This aligns with a previous study demonstrating that the degree of T2 inflammation at the start of a 48-week follow-up, assessed by FeNO or blood eosinophil count, was not predictive of T2 inflammation during exacerbations^29^. In contrast, we found that blood neutrophil count, CRP levels and the presence of an infiltrate on chest radiography were among the most important predictors of exacerbation severity. Neutrophilic inflammation, in both blood and tissue, is frequently associated with asthma exacerbations triggered by viral infections and has been linked to exacerbation severity^30,31^. These observations collectively suggest that pathogen-driven and non-eosinophilic pathways, such as inflammasome activation, interferon response, or Th-17 pathway upregulation play a more prominent role in determining exacerbation severity^32^. Exploring these mechanisms, could help develop targeted interventions.

Due to the retrospective design of our study, specific inflammatory pathways were not directly investigated. Furthermore, microbiological testing, including throat swabs and sputum cultures, was performed only in a subset of exacerbations. Nevertheless, we observed that rhinovirus and enterovirus infections were associated with ICU admissions. While rhinovirus infections typically cause mild symptoms in healthy individuals, it is known to induce more severe and prolonged episodes of airway dysfunction in asthma patients^33,34^. The role of enteroviruses in adult asthma exacerbations is less well characterized, but similar to rhinoviruses, they belong to the *Picornaviridae* family and have been linked to near-fatal asthma episodes^35^. These findings underscore the importance of further investigating non-eosinophilic pathways and viral triggers in asthma exacerbations.

Theophylline maintenance use was associated with increased exacerbation severity, including higher NEWS and ICU admission, and emerged as a key predictor of these measures and oxygenation efficiency. Patients on theophylline maintenance exhibited significantly higher NEWS (median 8, IQR 4–9 vs. 3, IQR 1–5, p < 0.001), worse oxygenation efficiency (median SpO_2_/FiO_2_ 460, IQR 362–480 vs. 475, IQR 460–490, p = 0.033), higher ICU admission rates (35.7% vs. 3.8%, p < 0.001), and greater need for mechanical ventilation (21.4% vs. 0.6%, p < 0.001) compared to non-users. However, despite a higher body mass index (BMI; 33.2 kg/m^2^ vs. 29.2 kg/m^2^, p = 0.023), the two groups were comparable in demographics, pulmonary function, and comorbidities. A review of the medical records revealed no evidence of cardiovascular, metabolic, or neurologic toxicity linked to theophylline. While theoretical concerns exist about theophylline causing muscle fatigue or impaired oxygenation efficiency due to enhanced respiratory drive and subtle ventilation-perfusion mismatch, these effects have not been consistently observed in clinical studies^36^. Therefore, it is most likely that theophylline users represent a subgroup with more severe or refractory disease.

The key strength of this study comprises the use of high-dimensional data from a larger cohort of asthma patients in a hospital care setting. Moreover, the associations between a wide range of clinical factors and five key outcomes related to asthma exacerbation severity were systematically investigated. Another notable strength is the use of advanced prediction modelling, incorporating both linear mixed models and LASSO regression. This dual approach not only identified independent factors associated with exacerbation severity but also leveraged a machine learning technique to select the most effective predictors. LASSO regression in particular enables efficient identification of key clinical predictors while minimizing overfitting, thereby enhancing the reliability and interpretability of our predictive models. A final major strength is the inclusion of multiple presentations per patient this allowed us to capture within-patient variability and better reflect the real-world complexity of asthma exacerbations. Despite variations in the course of consecutive exacerbations between individuals, patient-specific factors demonstrated a stronger predictive value for NEWS, oxygenation efficiency, and length of hospital stay than all other selected variables combined. This is demonstrated by the substantial differences between the marginal R^2^, which reflects the variability explained by fixed predictors, and the conditional R^2^, which accounts for both fixed predictors and patient-specific random effects. These findings highlight the significance of patient-specific variability and support the inclusion of multiple study visits per patient in future research designs.

This study suffers from a few limitations. Foremost, is the retrospective design which resulted in missing data for certain variables, which prevented their inclusion in the prediction models. Consequently, the predictive value of blood gas analyses, peak flow measurements during exacerbations, treatment adherence, exacerbation history, and detailed microbiological data could not be fully assessed. Notably, the missing data were not entirely random and were more common in cases of lower disease severity, potentially introducing bias. To maximize the number of exacerbations available for predictive modelling, we imputed missing values for six variables using the mean or median. Although the proportion of missing data was generally small, and imputation aimed to improve generalizability and increase statistical power, this approach may have underestimated variability and introduced bias in the LASSO models. The large discrepancy between the non-significant univariate association of eosinophil count and its predictive value in LASSO suggests this may have affected the results for oxygenation efficiency. No such discrepancies were observed for other variables. Additionally, the retrospective nature of the study limited the availability of potentially important data, particularly regarding inflammatory pathways and dynamic changes in biomarkers, which could have provided further mechanistic insights into exacerbation severity. We chose to limit data collection to the period before the national onset of the first wave of the COVID-19 pandemic to avoid potential confounding factors that could influence asthma exacerbation trends. However, this approach, combined with temporal shifts in treatment patterns, such as the increased adoption of biologic therapies (with 9.9% of patients already receiving biologics at the time of exacerbation) and the decreasing use of medications like theophylline, may impact the generalizability of our findings to contemporary clinical practice. These limitations should be considered when interpreting the study’s findings.

While our models incorporated a large number of variables, their predictive performance was moderate to poor, with a relatively low percentage of variability explained. The limited predictive accuracy for ICU admission may, in part, reflect the small number of ICU admissions in the dataset. Nevertheless, our findings do not support the routine clinical application of these prediction models. Instead, they underscore that the most important predictors of asthma exacerbation severity remain unidentified. We recommend that future research incorporate longitudinal monitoring of lung function and multi-compartment biomarker or omics-based approaches to better assess the dynamic nature of airway inflammation. Combining these data with advanced machine learning techniques could provide deeper insights into the complex mechanisms underlying exacerbation severity and ultimately lead to more personalized and effective management strategies for patients with asthma exacerbations.

## Conclusions

The presence of an infiltrate on chest radiography, pre-existing impaired lung function, elevated CRP levels and increased blood neutrophil count are the most consistent variables associated with exacerbation severity. However, despite using advanced machine learning, the predictive value of clinical and demographic parameters is limited, indicating the need to identify additional biomarkers or patient-specific traits to further improve tailored exacerbation management strategies.

## Supplementary Information

Supplementary Information.

## Data Availability

Pseudonymized patient data used in this study are available from the corresponding author (e.duijvelaar@amsterdamumc.nl) for other researchers when reuse conditions are met.

## Abbreviations

AUC: Area Under the Curve
BED: Beclomethasone dipropionate Equivalent Dosage
BMI: Body Mass Index
COPD: Chronic Obstructive Pulmonary Disease
CRP: C-reactive protein
CRSwNP: Chronic rhinosinusitis with nasal polyps
CRSsNP: Chronic rhinosinusitis without nasal polyps
CT: Computed Tomography
ED: Emergency Department
FDR: False Discovery Rate
FeNO: Fractional exhaled Nitric Oxide
FEV1: Forced Expiratory Volume in 1 s
FiO2: Fraction of inspired oxygen
FVC: Forced Vital Capacity
GERD: Gastroesophageal Reflux Disease
GINA: Global Initiative for Asthma
HMPV: Human Metapneumovirus
ICD: International Classification of Diseases
ICS: Inhalation Corticosteroids
ICU: Intensive Care Unit
IQR: Interquartile Range
LAMA: Long-Acting Muscarinic Antagonist
LASSO: Least Absolute Shrinkage and Selection operator
LMM: Linear Mixed Models
LTRA: Leukotriene receptor antagonist
NEWS: National Early Warning Score
NRM: Non-rebreathing mask
OCS: Oral Corticosteroid
OSAS: Obstructive Sleep Apnea Syndrome
PaCO2: Partial pressure of carbon dioxide
PaO2: Partial pressure of oxygen
PEF: Peak Expiratory Flow
ROC: Receiver Operating Characteristic
RSV: Respiratory Syncytial Virus
SpO2: Peripheral oxygen saturation
