# Correction: Machine learning reveals limited predictive value of clinical factors for asthma exacerbations

**DOI:** 10.1038/s41598-025-30215-x

**Published:** 2025-12-02

**Authors:** Erik Duijvelaar, Jack S. Gisby, Hanneke Coumou, Jeroen Hoogland, Bart Hilvering, Anirban Sinha, Marijke Amelink, Els J. M. Weersink

**Affiliations:** 1 Department of Pulmonary Medicine, Amsterdam UMC Location Vrije Universiteit Amsterdam, 1081 HV Amsterdam, The Netherlands; 2 William Harvey Research Institute, Queen Mary University of London, London, UK; 3 Department of Epidemiology and Data Science, Amsterdam UMC Location Vrije Universiteit, De Boelelaan 1089A, Amsterdam, The Netherlands; 4 Immunology Discovery Research, AbbVie Cambridge Research Center, Cambridge, MA USA; 5 Department of Pulmonary Medicine, Spaarne Gasthuis, Boerhaavelaan 22, Haarlem, the Netherlands

Correction to: *Scientific Reports* 10.1038/s41598-025-19056-w, published online 08 October 2025

The Competing interests section in the original version of this Article was incorrect.

“The authors declare no competing interests.”

now reads:

“Anirban Sinha is an employee of AbbVie and may hold stock or stock options in AbbVie. The other authors declare that they have no competing interests.”

The original Article has been corrected.

