# Supplementary material for: Machine learning reveals limited predictive value of clinical factors for asthma exacerbations
Source: Sci Rep. 2025 Oct 8;15:35198. doi: 10.1038/s41598-025-19056-w (PMC12508110; doi:10.1038/s41598-025-19056-w)
Supplement: Supplementary file 1 — Supplementary Information. [file 41598_2025_19056_MOESM1_ESM.docx]

Esupplement

Content

[eTable 1: Data dictionary belonging to the Source data 2](#_Toc205503914)

[eTable 2: conversion of oxygen suppletion to fraction of inspired oxygen 6](#_Toc205503915)

[eTable 3: Conversion factors used to calculate beclomethasone equivalent dosage (BED) 7](#_Toc205503916)

[eTable 4: Proportions of missing data for each variable 8](#_Toc205503917)

[eTable 5: Complete table of exacerbation characteristics 9](#_Toc205503918)

[eTable 6: Exacerbation and patients characteristics stratified by hospital admission 11](#_Toc205503919)

[eTable 7: Exacerbation and patients characteristics stratified by intensive care unit admission 14](#_Toc205503920)

[eTable 8: Complete table of associations between study characteristics and disease severity and clinical outcomes 17](#_Toc205503921)

[eTable 9: Variables selected for the prediction of hospital admission 21](#_Toc205503922)

[eTable 10: Variables selected for the prediction of intensive care unit admission 22](#_Toc205503923)

[eTable 11: Variables selected for the prediction of National Early Warning Score (NEWS) 23](#_Toc205503924)

[eTable 12: Variables selected for the prediction of the ratio of peripheral arterial oxygen saturation to the inspired fraction of oxygen (SpO2/FiO2) 24](#_Toc205503925)

[eTable 13: Variables selected for the prediction of length of hospital stay 25](#_Toc205503926)

[eTable 14: P-values of variables selected in the LASSO models in a subgroup of patients without comorbid COPD 26](#_Toc205503927)

# eTable 1: Data dictionary belonging to the Source data

| Variable name | Data type | Description |
| --- | --- | --- |
| Visit characteristics | | |
| Visits | Numerical (discrete) | Total number of emergency department visits |
| NumberofAE | Numerical (discrete) | Number of asthma exacerbations in the 12 months prior to presentation |
| TimesinceAE | Numerical (discrete) | Number of days since the last asthma exacerbation in the 12 months before presentation. |
| Durationsym | Numerical (discrete) | Duration of symptoms before visiting the emergency department (rounded off to the closest whole day) |
| Frequentexacerbator | Dichotomous (Yes/No) | Being a frequent exacerbator, defined as presented ≥2 times in the study time window (0=No, 1=Yes) |
| Date | Calendar date | Calendar date (dd/mm/yyyy) |
| Time | Numerical (discrete) | Time of presentation (rounded off to closest whole hour) |
| Season | categorical | Season of the year (Spring, Summer, Autumn and Winter) |
| Month | Numerical (discrete) | Month of year (January, February, March,...) |
| Patient characteristics | | |
| Age | Numerical (discrete) | Age rounded down to the closest whole year |
| Date of birth | Calendar date | Calendar date (dd/mm/yyyy) |
| Sex | Categorical | Biological sex (Male=0, Female=1) |
| Height | Numerical (continuous) | Height in centimeters |
| Weight | Numerical (continuous) | Weight in kilograms. |
| BMI | Numerical (continuous) | Body Mass Index (BMI) in kg/m2. |
| Race | Categorical | Ethnic race (Caucasian=0, Black=1, Asian or pacific islander=2, Hispanic=3, mixed=4, other=5) |
| smoking | Categorical | Smoking history of combustible tobacco (Never smoked=0, Past smoker=1, Current smoker=2). A past smoker is defined has having a history of more than 5 pack years. |
| Patient history | | |
| GERD | Dichotomous (Yes/No) | Having comorbid gastroesophageal reflux disease. Diagnostic Code in the 2 years prior presentation, chronic reflux symptoms, the use of PPIs, H2 blockers, or Antacids for reflux in the 2 years prior presentation). (1=yes, 0=no) |
| COPD | Dichotomous (Yes/No) | Having comorbid chronic obstructive pulmonary disease (Diagnostic code ever ) (1=yes, 0=no) |
| Rhinitis | Dichotomous (Yes/No) | Having comorbid chronic or allergic rhinitis (diagnostic code ever) (1=yes, 0=no) |
| CRSwNP | Dichotomous (Yes/No) | Having comorbid chronic rhinosinusitis with nasal polyps. (1=yes, 0=no) |
| CRSsNP | Dichotomous (Yes/No) | Having comorbid chronic rhinosinusitis without nasal polyps. (1=yes, 0=no) |
| bronchiectasis | Dichotomous (Yes/No) | Having comorbid bronchiectasis. (1=yes, 0=no) |
| OSAS | Dichotomous (Yes/No) | Having comorbid obstructive Sleep Apnea Syndrome, confirmed by polysomnography in the 2 years prior presentation or still requiring therapy. (1=yes, 0=no) |
| Psychosis | Dichotomous (Yes/No) | Experienced a psychosis in the 24 months prior presentation, or being diagnosed with schizophrenia. (1=yes, 0=no) |
| Anxiety disorder | Dichotomous (Yes/No) | Anxiety disorder in the 24 months prior presentation (1=yes, 0=no) |
| Depression | Dichotomous (Yes/No) | Depression in the 24 months prior presentation (1=yes, 0=no) |
| Urticaria | Dichotomous (Yes/No) | Having comorbid chronic or recurrent urticaria (History of, any code ever). A history of a single drug induced urticaria is not sufficient. (1=yes, 0=no) |
| NoofED | Numerical (discrete) | Number of hospital presentations in the 12 months prior to presentation due to asthma. This includes presentation to the emergency department of any hospital. |
| TimesinceED | Numerical (discrete) | Number of days since the last emergency department visit with an asthma exacerbation in the 12 months before presentation. |
| NoHA | Numerical (discrete) | Number of hospital admissions in the 12 months prior to presentation due to asthma. This could be an admission to any hospital. |
| TimesinceHA | Numerical (discrete) | Number of days since the last hospital admission with an asthma exacerbation in the 12 months before presentation. |
| Medication use | | |
| PreviousOCS | Numerical (discrete) | Number of times courses of systemic corticosteroids were used in the 12 months prior presentation. The use of oral corticosteroids as maintenance therapy does not add to the number of times systemic corticosteroids was used. Temporary dose increases to a dose commonly used to treat exacerbations are counted. |
| ICS | Dichotomous (Yes/No) | Current daily use of inhalation corticosteroids. (1=yes, 0=no) |
| ICSdosage | Numerical (discrete) | Current daily use of inhalation corticosteroids dosage (in beclomethasone equivalent doses in mcg) |
| ICScat | Categorical | Categorical inhalation corticosteroid dosage. (0= 0 mcg BED,1= low (100-600 mcg BED), 2= medium (601-1000 mcg BED), 3= high (1001-2000 mcg BED), 4= very high (>2000mcg BED)) |
| LAMA | Dichotomous (Yes/No) | Current daily use of inhaled long-acting muscarinic antagonists. (1=yes, 0=no) |
| LTRA | Dichotomous (Yes/No) | Current daily use of leukotriene receptor antagonists. (1=yes, 0=no) |
| Theophylline | Dichotomous (Yes/No) | Current daily use of theophylline. (1=yes, 0=no) |
| OCS | Dichotomous (Yes/No) | Current daily use of oral corticosteroid maintenance therapy. (1=yes, 0=no) |
| Biological | Dichotomous (Yes/No) | Current daily use of biological therapy. (0=no, 1=mepolizumab, 2=omalizumab, 3= reslizumab, 4= benralizumab, 5=dupilumab) |
| TreatAdh | Dichotomous (Yes/No) | Patient reported adequate treatment adherence. (1=yes, 0=no) |
| Lung function | | |
| Peakflow | Numerical (continuous) | Expiratory peak flow before nebulization with bronchodilators in L/min, measured during emergency department visit |
| PEFpred | Numerical (continuous) | Predicted expiratory peak flow, determined as follows:  Females: PEF =EXP((0,376*LN(age in years))-(0,012*age in years)-(58,8/height in cm)+5,63) Males: PEF = EXP((0,544*LN(age in years))-(0,0151*age in years)-(74,7/height in cm)+5,48) |
| PEFpercentage | Numerical (continuous) | Peak expiratory flow before nebulization as percentage of predicted = Peakflow/PEFpred |
| FVC | Numerical (continuous) | Forced Vital Capacity (FVC) before bronchodilation in the last lung function before presentation. When unavailable, the first lung function within 24 months after presentation was used instead. The lung function was not extracted if there was worsening of symptoms at the time of measurement or if there were technical issues that resulted in an unreliable result. |
| FVCpred | Numerical (continuous) | % Predicted Forced Vital Capacity (FVC) before bronchodilation in the last lung function before presentation. When unavailable, the first lung function within 24 months after presentation was used instead. The lung function was not extracted if there was worsening of symptoms at the time of measurement or if there were technical issues that resulted in an unreliable result. |
| FEV1 | Numerical (continuous) | Forced expiratory volume in the first second (FEV1) before bronchodilation in the last lung function before presentation. When unavailable, the first lung function within 24 months after presentation was used instead. The lung function was not extracted if there was worsening of symptoms at the time of measurement or if there were technical issues that resulted in an unreliable result. |
| FEV1pred | Numerical (continuous) | % Predicted forced expiratory volume in the first second (FEV1) before bronchodilation in the last lung function before presentation. When unavailable, the first lung function within 24 months after presentation was used instead. The lung function was not extracted if there was worsening of symptoms at the time of measurement or if there were technical issues that resulted in an unreliable result. |
| FEV1revPer | Numerical (continuous) | % reversibility of the forced expiratory volume in the first second (FEV1) in the last lung function before presentation. When unavailable, the first lung function within 24 months after presentation was used instead. The lung function was not extracted if there was worsening of symptoms at the time of measurement or if there were technical issues that resulted in an unreliable result. |
| FEV1rev | Dichotomous (Yes/No) | Reversibility of the forced expiratory volume in the first second (FEV1) >200mL and >12% in the last lung function before presentation. When unavailable, the first lung function within 24 months after presentation was used instead. The lung function was not extracted if there was worsening of symptoms at the time of measurement or if there were technical issues that resulted in an unreliable result. |
| FEV1/FVC | Numerical (continuous) | Forced expiratory volume in the first second (FEV1)/ Forced Vital Capacity (FVC) before bronchodilation in the last lung function before presentation. When unavailable, the first lung function within 24 months after presentation was used instead. The lung function was not extracted if there was worsening of symptoms at the time of measurement or if there were technical issues that resulted in an unreliable result. |
| Allergy status | | |
| Allergy | Dichotomous (Yes/No) | Allergic sensitization to aeroallergens, ideally confirmed by either a radioallergosorbent test (RAST) or skin prick test. If there are no specific tests done, the allergy status should be entered by a physician in the allergy section of the patient record. (1=yes, 0=no) |
| Aspergillus | Dichotomous (Yes/No) | Allergic sensitization to Aspergillus Fumigatus (m3) confirmed by either a radioallergosorbent (RAST) or skin prick test. (1=yes, 0=no) |
| Peripheral blood markers | | |
| Eosinophil | Numerical (continuous) | Blood eosinophil count *10^9/L on presentation |
| Neutrophil | Numerical (continuous) | Blood neutrophil count *10^9/L on presentation |
| Leukocyte | Numerical (continuous) | Blood leukocyte count *10^9/L on presentation |
| Higheos | Dichotomous (Yes/No) | Blood eosinophil count on presentation ≥ 0.40 *10^9/L. (1=yes, 0=no) |
| CRP | Numerical (continuous) | C-reactive protein (mg/L) on presentation |
| Provocative factors | | |
| Flu | Categorical | Flu like symptoms = one or a combination of the following symptoms: sore throat, headache, muscle aches and runny or stuffy nose. |
| Infiltrate | Dichotomous (Yes/No) | Infiltrate on X-Ray or CT-scan confirmed by a radiologist. (1=yes, 0=no) |
| Allergenexposure | Dichotomous (Yes/No) | Allergen exposure played a significant factor as the cause of the exacerbation according to the patient. (1=yes, 0=no) |
| Nonspecifictrigger | Dichotomous (Yes/No) | Non-specific trigger exposure played a significant factor as the cause of the exacerbation according to the patient. (1=yes, 0=no) |
| Occupationalexposure | Dichotomous (Yes/No) | Irritant or occupational exposure played a significant factor as the cause of the exacerbation according to the patient. (1=yes, 0=no) |
| Anyvirus | Dichotomous (Yes/No) | Any viral infection on presentation (DNA/RNA detected) by nasal and/or throat swab. (1=yes, 0=no) |
| Rhinovirus | Dichotomous (Yes/No) | Rhinovirus RNA detected on presentation by nasal and/or throat swab. (1=yes, 0=no) |
| Influenza | Dichotomous (Yes/No) | Influenza RNA detected on presentation by nasal and/or throat swab. (1=yes, 0=no) |
| HMPV | Dichotomous (Yes/No) | Human metapneumovirus (HMPV) RNA detected on presentation by nasal and/or throat swab. (1=yes, 0=no) |
| RSV | Dichotomous (Yes/No) | Respiratory syncytial virus (RSV) DNA detected on presentation by nasal and/or throat swab. (1=yes, 0=no) |
| Parainfluenza | Dichotomous (Yes/No) | Parainfluenza RNA detected on presentation by nasal and/or throat swab. (1=yes, 0=no) |
| Corona | Dichotomous (Yes/No) | Coronavirus (except for SARS-CoV-1 or SARS-CoV-2 variants) RNA detected on presentation by nasal and/or throat swab. (1=yes, 0=no) |
| Enterovirus | Dichotomous (Yes/No) | Enterovirus RNA detected on presentation by nasal and/or throat swab. (1=yes, 0=no) |
| Anybacteria | Dichotomous (Yes/No) | Any bacterial infection cultured in sputum or positive urinary antigen test. (1=yes, 0=no) |
| Streptococ | Dichotomous (Yes/No) | Streptococcus Pneumoniae infection (confirmed with either urinary antigen Test or sputum culture). (1=yes, 0=no) |
| Moraxella Catarrhalis | Dichotomous (Yes/No) | Moraxella Catarrhalis infection (confirmed by sputum culture). (1=yes, 0=no) |
| Hemophilus | Dichotomous (Yes/No) | Hemophilus Influenzae infection (confirmed by sputum culture). (1=yes, 0=no) |
| S.Aureus | Dichotomous (Yes/No) | Staphylococcus Aureus infection (confirmed by sputum culture). (1=yes, 0=no) |
| Legionella | Dichotomous (Yes/No) | Legionella infection (confirmed by sputum culture or urinary Antigen Test). (1=yes, 0=no) |
| AspergillusFumigatus | Dichotomous (Yes/No) | Aspergillus Fumigatus infection (confirmed by sputum culture). (1=yes, 0=no) |
| Pseudomonas | Dichotomous (Yes/No) | Pseudomonas aeruginosa infection (confirmed by sputum culture). (1=yes, 0=no) |
| Klebsiella | Dichotomous (Yes/No) | Klebsiella Oxytoca infection (confirmed by sputum culture). (1=yes, 0=no) |
| Clinical outcome and disease severity | | |
| pH | Numerical (continuous) | Arterial blood pH, only when drawn from an artery. Not recorded if there is a suspicion of venous blood collection, i.e. discrepancy with pulsoximetry. |
| PaCO2 | Numerical (continuous) | PaCO2 in arterial blood (kPa), only when drawn from an artery. Not recorded if there is a suspicion of venous blood collection. |
| PaO2 | Numerical (continuous) | PaO2 in arterial blood (kPa), only when drawn from an artery. Not recorded if there is a suspicion of venous blood collection. |
| O2 | Numerical (continuous) | Oxygen saturation in % in arterial blood. Not recorded if there is a suspicion of venous blood collection. |
| SpO2 | Numerical (continuous) | Peripheral oxygen saturation in % at presentation, measured with pulse oximetry |
| FiO2 | Numerical (continuous) | Fraction of inspired oxygen at presentation (see supplementary eTable 2) |
| Hypercapnia | Dichotomous (Yes/No) | Hypercapnia defined as PaCO2 > 6.0 kPa (1=yes, 0=no) |
| NEWS | Numerical (discrete) | National Early Warning Score (NEWS) |
| Admission | Dichotomous (Yes/No) | Hospital admission. Spending at least one night in the hospital when the patient needs to be transferred to a different hospital due to logistic reasons. (1=yes, 0=no) |
| LOS | Numerical (discrete) | Length of hospital stay in days (per 24hours) rounded off to closest whole number |
| ICU | Dichotomous (Yes/No) | Admission to the intensive care unit. (1=yes, 0=no) |
| Intubation | Dichotomous (Yes/No) | Requiring invasive ventilation.(1=yes, 0=no) |
| Mortality | Dichotomous (Yes/No) | Mortality due to the asthma exacerbation or its complications. (1=yes, 0=no) |

# eTable 2: conversion of oxygen suppletion to fraction of inspired oxygen

|  |  | Fraction of inspired oxygen (FiO2) |
| --- | --- | --- |
| Room Air |  | 20% |
| Nasal Canula | 1L/min | 22% |
|  | 2L/min | 24% |
|  | 3L/min | 26% |
|  | 4L/min | 28% |
|  | 5L/min | 30% |
|  | 6L/min | 32% |
| Mask Not Specified | 2L/min | 24% |
|  | 3L/min | 26% |
|  | 4L/min | 28% |
|  | 5L/min | 30% |
|  | 6L/min | 31% |
|  | 7L/min | 33% |
|  | 8L/min | 35% |
|  | 9L/min | 38% |
|  | 10L/min | 40% |
| Venturi Mask | 2L/min | 24% |
|  | 4L/min | 28% |
|  | 6L/min | 31% |
|  | 8L/min | 35% |
|  | 10L/min | 40% |
|  | 12L/min | 40% |
|  | 15L/min | 60% |
| Non-rebreathing mask | 8L/min | 50% |
|  | 9L/min | 55% |
|  | 10L/min | 60% |
|  | 11L/min | 65% |
|  | 12L/min | 70% |
|  | 13L/min | 75% |
|  | 14L/min | 80% |
|  | 15L/min | 85% |
| high flow nasal oxygen |  | FiO2 is given |
| Intubation |  | FiO2 is given |

# eTable 3: Conversion factors used to calculate beclomethasone equivalent dosage (BED)

| Inhalation corticosteroid | BED (mcg) | Factor |
| --- | --- | --- |
| Beclomethasone dipropionate (CFC) | 1000 mcg | x 1 |
| Beclomethasone dipropionate (HFA) | 400 mcg | x 2,5 |
| Budesonide | 800 mcg | x 1,25 |
| Ciclesonide | 320 mcg | x 3,125 |
| Fluticasone furoate | 100 mcg | x 10 |
| Fluticasone propionate | 500 mcg | x 2 |

CFC= chlorofluorocarbon, HFA = hydrofluoroalkanes

# eTable 4: Proportions of missing data for each variable

| Variable name | Data missing |
| --- | --- |
| Allergy* | 4 (1.1%) |
| Any bacteria | 503 (78.1%) |
| Any virus | 509 (79.0%) |
| Aspergillus Fumigatus* | 219 (59.7%) |
| Blood eosinophil count | 86 (13.4%) |
| Blood neutrophil count | 99 (15.4%) |
| BMI | 125 (19.4%) |
| CRP | 70 (10.9%) |
| Duration of symptoms | 6 (0.9%) |
| Ethnicity* | 29 (7.9%) |
| FEV1 | 117 (18.2%) |
| FEV1 % of predicted | 112 (17.4%) |
| FEV1 % reversible | 280 (43.5%) |
| FEV1 reversible | 279 (43.3%) |
| FEV1/FVC | 112 (17.4%) |
| FVC | 117 (18.2%) |
| FVC % of predicted | 117 (18.2%) |
| Height* | 88 (24.0%) |
| Hypercapnia | 266 (41.3%) |
| Infiltrate on X-ray or CT-scan | 68 (10.6%) |
| Length of hospital stay | 15 (2.3%) |
| Leukocyte | 59 (9.2%) |
| NEWS | 7 (1.1%) |
| Oxygen saturation in arterial blood | 267 (41.5%) |
| PaCO2 in arterial blood | 265 (41.1%) |
| PaO2 in arterial blood | 265 (41.1%) |
| PaO2/FiO2 | 265 (41.1%) |
| Peak Expiratory Flow | 475 (73.8%) |
| Peak Expiratory Flow % of predicted | 504 (78.3%) |
| pH in arterial blood | 265 (41.1%) |
| Saturation of Peripheral Oxygen (SpO2) | 6 (0.9%) |
| Smoking status* | 15 (4.1%) |
| SpO2/FiO2 | 6 (0.9%) |
| Time since asthma exacerbation | 409 (63.5%) |
| Time since emergency department visit | 417 (64.8%) |
| Time since hospital admission | 498 (77.3%) |
| Treatment adherence | 595 (92.4%) |
| Weight | 121 (18.8%) |

The proportions of missing data were computed across all exacerbations. However, for variables marked with an *, the proportion of missing data was computed using the number of patients as the denominator. BMI = Body Mass Index, , CRP = C-reactive protein, FEV1 = Forced Expiratory Volume in 1 second, FiO2 = Fraction of inspired oxygen, FVC= Forced Vital Capacity, NEWS = National Early Warning Score, OCS = Oral Corticosteroids, PaCO2 = Partial pressure of carbon dioxide, PaO2 = Partial pressure of Oxygen, PEF = Peak Expiratory Flow.

# eTable 5: Complete table of exacerbation characteristics

| Characteristic | All exacerbations N=644 |
| --- | --- |
| Exacerbation history | |
| Time since previous asthma exacerbation (days) N=235 | 92 [38, 211] |
| Number of asthma exacerbations 12 months prior | 0 [0, 1] |
| Time since previous emergency department visit due to an asthma exacerbation (days) N=227 | 84 [32, 208] |
| Number of emergency department visits 12 months prior | 0 [0, 1] |
| Time since previous hospital admission (days) N=146 | 102 [37, 209] |
| Number of hospital admissions 12 months prior | 0 [0, 0] |
| Duration of symptoms (days) N=634 | 4 [2, 14] |
| Season | |
| Spring | 145 (22.5%) |
| Summer | 149 (23.1%) |
| Autumn | 150 (23.3%) |
| Winter | 200 (31.1%) |
| Medication use | |
| Daily maintenance use |  |
| Inhalation Corticosteroid | 486 (75.5%) |
| Inhalation Corticosteroid dosage (in mcg BED) | 1000 [0, 2000] |
| Long-Acting Muscarinic Antagonist | 176 (27.3%) |
| Leukotriene Receptor Antagonists | 98 (15.2%) |
| Theophylline | 14 (2.2%) |
| corticosteroid maintenance therapy | 159 (24.7%) |
| Inhalation Corticosteroid category |  |
| 0-200 mcg BED | 162 (25.2%) |
| 201-500 mcg BED | 53 (8.2%) |
| 501-1000 mcg BED | 119 (18.5%) |
| >1000 mcg BED | 310 (48.1%) |
| Biological |  |
| Mepolizumab | 35 (5.4%) |
| Omalizumab | 22 (3.4%) |
| Reslizumab | 6 (0.9%) |
| Benralizumab | 0 (0%) |
| Dupilumab | 1 (0.2%) |
| Treatment adherence N=49 |  |
| No | 32 (65.3%) |
| Yes | 17 (34.7%) |
| Number of OCS courses used in last 12 months | 0 [0, 1] |
| Blood markers | |
| Eosinophil (x 10^9/L) N=558 | 0.15 [0.04, 0.39] |
| Neutrophil (x 10^9/L) N=545 | 7.02 ± 3.53 |
| Leukocyte (x 10^9/L) N=585 | 10.2 ± 3.9 |
| CRP (mg/L) N=574 | 5.9 [2.2, 16.5] |
| Patient reported triggers | |
| Flu like symptoms | 339 (52.6) |
| Allergen exposure | 51 (7.9%) |
| Non-specific trigger | 61 (9.5%) |
| Occupational exposure | 2 (0.3%) |
| Lung function at presentation | |
| Peak expiratory flow (L/min) N=355 | 241 ± 127 |
| Peak expiratory flow % of predicted N=355 | 45.0 ± 21.1 |
| Lung function prior presentation | |
| FVC (L) N=270 | 3.38 ± 1.12 |
| FVC % of predicted N=270 | 92.2 ± 18.9 |
| FEV1 (L) N=270 | 2.35 ± 0.90 |
| FEV1 % of predicted N=265 | 76.7 ± 21.0 |
| FEV1 % reversibility N=193 | 6.91 ± 8.66 |
| FEV1 reversible N=193 | 89 (24.4%) |
| FEV1/FVC N=275 | 0.68 ± 0.13 |
| Radiologic findings | |
| Infiltrate on X-ray or CT-scan N = 576 | 65 (11.3%) |
| Viral infection N = 135 | |
| Any viral infection | 84 (62.2%) |
| Rhinovirus | 33 (24.4%) |
| Influenza | 29 (21.3%) |
| Human Metapneumovirus | 8 (5.9%) |
| Respiratory Syncytial Virus | 6 (4.4%) |
| Parainfluenza | 4 (3.0%) |
| Coronavirus* | 9 (6.7%) |
| Enterovirus | 2 (1.5%) |
| Bacterial infection N=140 | |
| Any bacterial infection | 23 (16.3%) |
| Streptococcus Pneumoniae | 4 (2.9%) |
| Moraxella Catarrhalis | 2 (1.4%) |
| Hemophilus Influenzae | 15 (10.7%) |
| Staphylococcus Aureus | 2 (1.4%) |
| Aspergillus Fumigatus | 6 (4.3%) |
| Klebsiella Oxytoca | 2 (1.4%) |
| Pseudomonas aeruginosa | 2 (1.4%) |
| Arterial blood gas and oxygenation | |
| pH in arterial blood N=379 | 7.46 ± 0.08 |
| PaCO2 in arterial blood (kPa) N=379 | 4.62 ± 1.44 |
| PaO2 in arterial blood (kPa) N=379 | 11.8 ± 4.7 |
| Oxygen saturation (%) in arterial blood N=379 | 94.2 ± 3.2 |
| Hypercapnia in arterial blood N=379 | 15 (4.0%) |
| SpO2 (%) N=638 | 96 [93, 98] |
| FiO2 (%) | 20 [20, 20] |
| SpO2/FiO2 N=638 | 475 [460, 490] |
| PaO2/FiO2 (mmHg) N=265 | 399 ± (141) |
| Clinical outcome and disease severity | |
| NEWS N=637 | 3 [1, 5] |
| Admission | 279 (43.3%) |
| Length of hospital stay (days) N=629 | 0 [0, 3] |
| Length of hospital stay in admitted patients (days) N=264 | 3 [2, 6] |
| Intensive care admission | 29 (4.5%) |
| Intubation | 7 (1.1%) |
| Mortality | 0 (0%) |

The total number of exacerbations used for analysis was N=644, unless stated otherwise. Data is presented as proportion (percentage), mean ± SD or median [IQR]. BED = Beclomethasone Equivalent Dose, CRP = C-reactive protein, CT = Computed Tomography, FEV1 = Forced Expiratory Volume in 1 second, FiO2 = Fraction of inspired oxygen , FVC= Forced Vital Capacity, mcg = micrograms, NEWS = National Early Warning Score, PaCO2= partial pressure of carbon dioxide, PaO2= partial pressure of oxygen, SpO2 = Saturation of Peripheral Oxygen. *Excluding Severe acute respiratory syndrome coronavirus (SARS-COV) variants 1 and 2.

# eTable 6: Exacerbation and patients characteristics stratified by hospital admission

| Characteristic | Not admitted N = 365 | Admitted N = 279 | p-value |
| --- | --- | --- | --- |
| Patient characteristics | | | |
| Age | 43.0 [29.0, 55.0] | 46.0 [31.0, 58.0] | 0.212 |
| Female Sex | 221 (60.5%) | 190 (68.1%) | 0.058 |
| Height (cm) N=542 | 167.5 ± 10.1 | 167.5 ± 10.5 | 0.976 |
| Weight (kg) N=523 | 81.5 ± 17.5 | 83.1 ± 18.2 | 0.312 |
| BMI (kg/m2) N=519 | 29.1 ± 6.0 | 29.8 ± 6.5 | 0.268 |
| Ethnicity N=609 |  |  | 0.579 |
| Caucasian | 179 (52.8%) | 161 (59.6%) |  |
| Black | 65 (19.2%) | 45 (16.7%) |  |
| Asian or pacific islander | 20 (5.9%) | 11 (4.1%) |  |
| Hispanic | 7 (2.1%) | 3 (1.1%) |  |
| Mixed | 21 (6.2%) | 16 (5.9%) |  |
| Other | 47 (13.9%) | 34 (12.6%) |  |
| Smoking status N=628 |  |  | 0.371 |
| Never smoked | 197 (56.1%) | 146 (53.1%) |  |
| Past smoker | 86 (24.5%) | 81 (29.5%) |  |
| Current smoker | 68 (19.4%) | 48 (17.5%) |  |
| Comorbidities |  |  |  |
| GERD | 61 (16.7%) | 50 (17.9%) | 0.766 |
| COPD | 58 (15.9%) | 51 (18.3%) | 0.487 |
| rhinitis | 141 (38.6%) | 118 (42.3%) | 0.391 |
| CRSwNP | 39 (10.7%) | 40 (14.3%) | 0.201 |
| CRSsNP | 36 (9.9%) | 33 (11.8%) | 0.503 |
| Bronchiectasis | 28 (7.7%) | 23 (8.2%) | 0.905 |
| OSAS | 36 (9.9%) | 32 (11.5%) | 0.598 |
| Urticaria | 22 (6.0%) | 14 (5.0%) | 0.704 |
| Allergy |  |  |  |
| Aeroallergens N=639 | 235 (68.1%) | 187 (69.5%) | 0.777 |
| Aspergillus Fumigatus N=310 | 38 (24.4%) | 23 (14.9%) | 0.052 |
| Exacerbation Characteristics | | | |
| Total emergency department visits during study time period | 2.0 [1.0, 4.0] | 2.0 [1.0, 5.0] | **0.009** |
| Time since previous asthma exacerbation (days) N=235 | 94.0 [43.0, 216.0] | 92.0 [32.0, 201.2] | 0.269 |
| Number of asthma exacerbations 12 months prior | 0.71 ± 1.46 | 1.03 ± 1.83 | **0.014** |
| Time since previous emergency department visit due to an asthma exacerbation (days) N=227 | 85.0 [41.0, 216.0] | 81.0 [25.5, 198.50] | 0.148 |
| Number of emergency department visits 12 months prior | 0.7 ± 1.3 | 1.0 ± 1.8 | **0.007** |
| Time since previous hospital admission (days) N=146 | 118.0 [72.5, 244.5] | 92.0 [30.5, 190.5] | **0.017** |
| Number of hospital admissions 12 months prior | 0.3 ± 0.8 | 0.6 ± 1.2 | **0.001** |
| Duration of symptoms (days) N=634 | 4.0 [2.0, 12.0] | 4.0 [2.0, 4.0] | 0.804 |
| Season |  |  | 0.895 |
| Spring | 80 (21.9%) | 65 (23.3%) |  |
| Summer | 84 (23.0%) | 65 (23.3%) |  |
| Autumn | 89 (24.4%) | 61 (21.9%) |  |
| Winter | 112 (30.7%) | 88 (31.5%) |  |
| Medication use | | | |
| Daily maintenance use |  |  |  |
| Inhalation Corticosteroid | 266 (73.1%) | 216 (78.3%) | 0.157 |
| Inhalation Corticosteroid dosage (in mcg BED) | 2000[1000, 3000] | 2000[1000, 3000] | 0.459 |
| Long-Acting Muscarinic Antagonist | 79 (21.6%) | 97 (34.8%) | **<0.001** |
| Leukotriene Receptor Antagonists | 55 (15.1%) | 43 (15.4%) | 0.992 |
| Theophylline | 4 (1.1%) | 10 (3.6%) | 0.061 |
| corticosteroid maintenance therapy | 70 (19.2%) | 89 (31.9%) | <0.001 |
| Inhalation Corticosteroid category |  |  | 0.180 |
| 0-200 mcg BED | 99 (27.1%) | 63 (22.6%) |  |
| 201-500 mcg BED | 31 (8.5%) | 22 (7.9%) |  |
| 501-1000 mcg BED | 73 (20.0%) | 46 (16.5%) |  |
| >1000 mcg BED | 162 (44.4%) | 148 (53.0%) |  |
| Biological |  |  | 0.177 |
| None | 327 (89.6%) | 253 (90.7%) |  |
| Mepoluzimab | 18 (4.9%) | 17 (6.1%) |  |
| Omalizumab | 17 (4.7%) | 5 (1.8%) |  |
| Reslizumab | 2 (0.5%) | 4 (1.4%) |  |
| Dupilumab | 1 (0.3%) | 0 (0.0%) |  |
| Treatment adherence N=49 | 8 (27.6%) | 9 (45.0%) | 0.340 |
| Number of OCS courses used in last 12 months |  |  |  |
| Blood markers | | | |
| Eosinophil (x 10^9/L) N=558 | 0.17 [0.07, 0.39] | 0.14 [0.02, 0.39] | 0.106 |
| Neutrophil (x 10^9/L) N=545 | 6.1 [4.5, 8.4] | 6.7 [4.6, 9.4] | **0.082** |
| Leukocyte (x 10^9/L) N=585 | 9.4 [7.6,0.082 | 10.1 [7.4, 12.6] | 0.155 |
| CRP (mg/l) N=574 | 5.4 [1.9, 13.8] | 6.5 [2.8, 19.0] | 0.075 |
| Lung function at presentation | | | |
| Peakflow (L/min) N=355 | 266.27 ± 126.03 | 203.03 ± 119.96 | **0.001** |
| PEF % of predicted N=355 | 48.2 ± 19.2 | 40.9 ± 22.9 | **0.042** |
| Lung function prior presentation | | | |
| FVC (L) N=270 | 3.4 ± 1.1 | 3.4 ± 1.2 | 0.714 |
| FVC % of predicted N=270 | 92.0 ± 18.3 | 92.5 ± 19.5 | 0.788 |
| FEV1 (L) N=270 | 2.3 [1.7, 3.0] | 2.2 [1.6, 2.8] | 0.066 |
| FEV1 % of predicted N=265 | 78.6 ± 20.4 | 74.5 ± 21.4 | **0.025** |
| FEV1 % reversibility N=193 | 5.0 [2.0, 11.0] | 5.0 [2.0, 11.0] | 0.949 |
| FEV1 reversible N=193 | 49 (25.4%) | 40 ( 23.3%) | 0.725 |
| FEV1/FVC N=275 | 0.70 ± 0.13 | 0.66 ± 0.13 | **0.002** |
| Patient reported triggers | | | |
| Flu like symptoms | 190 (52.2%) | 149 (53.4%) | 0.823 |
| Allergen exposure | 33 (9.1%) | 18 (6.5%) | 0.285 |
| Non-specific trigger | 35 (9.6%) | 26 (9.3%) | 1.000 |
| Occupational exposure |  |  |  |
| Radiologic findings | | | |
| Infiltrate | 22 (7.1%) | 43 (16.1%) | **0.001** |
| Viral infection N = 135 | | | |
| Any viral infection | 30 (61.2%) | 54 (62.8%) | 1.000 |
| Rhinovirus | 10 (20.4%) | 23 (26.7%) | 0.538 |
| Influenza | 11 (22.4%) | 18 (20.7%) | 0.982 |
| Human Metapneumovirus | 2 (4.1%) | 6 (7.0%) | 0.760 |
| Respiratory Syncytial Virus | 2 (4.1%) | 4 (4.7%) | 1.000 |
| Parainfluenza | 4 (8.2%) | 0 (0.0%) | **0.031** |
| Coronavirus* | 4 (8.2%) | 5 (5.8%) | 0.867 |
| Enterovirus | 0 (0.0%) | 2 (2.3%) | 0.738 |
| Bacterial infection N=140 | | | |
| Any bacterial infection | 9 (17.3%) | 14 (15.7%) | 0.993 |
| Streptococcus Pneumoniae | 3 (5.8%) | 1 (1.1%) | 0.287 |
| Moraxella Catarrhalis | 1 (1.9%) | 1 (1.1%) | 1.000 |
| Hemophilus Influenzae | 4 (7.7%) | 11 (12.5%) | 0.545 |
| Staphylococcus Aureus | 1 (1.9%) | 1 (1.1%) | 1.000 |
| Aspergillus Fumigatus | 4 (7.8%) | 2 (2.3%) | 0.261 |
| Klebsiella Oxytoca | 0 (0.0%) | 2 (2.3%) | 0.720 |
| Pseudomonas aeruginosa | 0 (0.0%) | 2 (2.3%) | 0.720 |
| Arterial blood gas and oxygenation | | | |
| pH in arterial blood N=379 | 7.4 [7.4, 7.5] | 7.4 [7.4, 7.5] | 0.434 |
| PaCO2 in arterial blood N=379 | 4.6 [4.1, 5.0] | 4.6 [4.1, 5.2] | 0.211 |
| PaO2 in arterial blood N=379 | 10.8 [9.4, 12.5] | 9.9 [8.7, 13.4] | 0.124 |
| Oxygen saturation (%) in arterial blood N=379 | 95.0 [93.4, 96.2] | 94.4 [91.8, 96.8] | 0.410 |
| Hypercapnia in arterial blood N=379 | 1 (0.6%) | 14 (6.3%) | **0.011** |
| SpO2 N=638 | 96.0 [94.0, 98.0] | 94.0 [92.0, 97.0] | **<0.001** |
| FiO2 | 20.00 [20.00, 20.00] | 20.00 [20.00, 20.00] | **<0.001** |
| SpO2/FiO2 N=638 | 476.47 ± 25.72 | 434.07 ± 82.28 | **<0.001** |
| PaO2/FiO2 (mmHg) N=265 |  |  |  |
| Disease severity | | | |
| NEWS N=622 | 2.0 [1.0, 4.0] | 4.0 [2.0, 6.0] | **<0.001** |

The total number of exacerbations used to describe proportions was N=644, unless stated otherwise. Data is presented as proportion (percentage), mean ± SD or median [IQR]. BED = Beclomethasone Equivalent Dose, CRP = c-reactive protein, CT = Computed Tomography, FEV1 = Forced Expiratory Volume in 1 second, FiO2 = Fraction of inspired oxygen , FVC= Forced Vital Capacity, HMPV = Human Metapneumovirus, ICS = Inhalation Corticosteroid, ICU = Intensive Care Unit, LAMA = Long Acting Muscarinic Antagonist, LTRA = Leukotriene Receptor Antagonists, NEWS = National Early Warning Score, OCS = Oral Corticosteroids, PaCO2= partial pressure of carbon dioxide, PaO2= partial pressure of oxygen PEF = Peak Expiratory Flow, RSV = Respiratory Syncytial Virus, SpO2 = Saturation of Peripheral Oxygen. *Excluding Severe acute respiratory syndrome coronavirus (SARS-COV) variants 1 and 2.

# eTable 7: Exacerbation and patients characteristics stratified by intensive care unit admission

| Variable | Not admitted to ICU  N=615 | Admitted to ICU  N=29 | p-value |
| --- | --- | --- | --- |
| Patient characteristics | | | |
| Total visits | 2.00 [1.00, 4.00] | 2.00 [1.00, 4.00] | 0.579 |
| Multiple (≥2) exacerbator | 377 (61.3%) | 17 (58.6%) | 0.925 |
| Age | 44.81 ± 17.40 | 40.21 ± 16.13 | 0.163 |
| Sex = female | 392 (63.7%) | 19 (65.5%) | 1.000 |
| Weight (kg) N = 266 | 81.93 ± 17.56 | 88.32 ± 22.00 | 0.070 |
| Height (cm) N = 279 | 167.48 ± 10.35 | 168.34 ± 8.34 | 0.667 |
| BMI (kg/m2) N = 264 | 29.33 ± 6.17 | 31.14 ± 7.56 | 0.143 |
| Ethnicity |  |  | 0.310 |
| Caucasian | 322 (55.4%) | 18 (64.3%) |  |
| Black | 107 (18.4%) | 3 (10.7%) |  |
| Asian or pacific islander | 30 (5.2%) | 1 (3.6%) |  |
| Hispanic | 10 (1.7%) | 0 (0.0%) |  |
| Mixed | 33 (5.7%) | 4 (14.3%) |  |
| Other | 79 (13.6%) | 2 (7.1%) |  |
| Smoking N = 352 |  |  | 0.973 |
| Never smoked | 328 (54.8%) | 15 (53.6%) |  |
| Past smoker | 159 (26.6%) | 8 (28.6%) |  |
| Current smoker | 111 (18.6%) | 5 (17.9%) |  |
| Comorbidities |  |  |  |
| GERD | 106 (17.2%) | 5 (17.2%) | 1.000 |
| COPD | 105 (17.1%) | 4 (13.8%) | 0.836 |
| Rhinitis | 248 (40.3%) | 11 (37.9%) | 0.950 |
| CRSwNP | 77 (12.5%) | 2 (6.9%) | 0.540 |
| CRSsNP | 64 (10.4%) | 5 (17.2%) | 0.392 |
| bronchiectasis | 51 (8.3%) | 0 (0.0%) | 0.206 |
| OSAS | 64 (10.4%) | 4 (13.8%) | 0.787 |
| Urticaria | 35 (5.7%) | 1 (3.4%) | 0.920 |
| Allergies N = 363 |  |  |  |
| Aeroallergens | 407 (69.3%) | 15 (55.6%) | 0.194 |
| Aspergillus Fumigatus | 60 (20.7%) | 1 (5.0%) | 0.157 |
| Blood values | | | |
| Eosinophil (x10^9/L) N=558 | 0.15 [0.04, 0.40] | 0.20 [0.05, 0.38] | 0.698 |
| Neutrophil (x10^9/L) N=545 | 6.20 [4.47, 8.62] | 9.94 [6.52, 12.47] | **<0.001** |
| Leukocyte (x10^9/L) N=585 | 9.60 [7.50, 12.03] | 12.30 [9.20, 16.00] | **0.004** |
| Lung function | | | |
| Peakflow (L/Min) N=355 | 243.27 ± 127.05 | 140.00 ± 93.81 | 0.109 |
| PEF % of predicted N=355 | 45.31 ± 21.01 | 30.74 ± 23.85 | 0.238 |
| FVC % of predicted N=270 | 92.15 ± 18.91 | 93.63 ± 18.52 | 0.692 |
| FEV1 % of predicted N=265 | 76.70 (20.80) | 76.46 ± 24.04 | 0.953 |
| FEV1 % reversible N=193 | 5.00 [2.00, 11.00] | 7.50 [2.25, 10.25] | 0.771 |
| FEV1 reversible N=193 | 85 (24.8%) | 4 (18.2%) | 0.658 |
| FEV1/FVC N=275 | 0.68 ± 0.13 | 0.67 ± 0.14 | 0.789 |
| Exacerbation history | | | |
| Time since asthma exacerbation (days) N=235 | 92.00 [38.75, 210.00] | 36.00 [22.50, 192.50] | 0.463 |
| Time since emergency department visit (days) N=227 | 84.50 [32.00, 207.25] | 36.00 [22.50, 192.50] | 0.621 |
| Time since hospital admission (days) N=146 | 103.00 [39.00, 209.00] | 27.00 [18.00, 36.00] | 0.124 |
| Number of emergency department visits 12 months prior | 0.00 [0.00, 1.00] | 0.00 [0.00, 0.00] | 0.132 |
| Number of hospital admissions 12 months prior | 0.00 [0.00, 0.00] | 0.00 [0.00, 0.00] | 0.406 |
| Number of asthma exacerbations 12 months prior | 0.00 [0.00, 1.00] | 0.00 [0.00, 0.00] | 0.117 |
| Duration of symptoms (days) | 4.00 [2.00, 14.00] | 1.00 [0.00, 3.00] | **<0.001** |
| Season |  |  | **0.007** |
| Autumn | 136 (22.1%) | 14 (48.3%) |  |
| Spring | 143 (23.2%) | 2 (6.9%) |  |
| Summer | 144 (23.4%) | 5 (17.2%) |  |
| Winter | 192 (31.2%) | 8 (27.6%) |  |
| Medication use | | | |
| OCS used in last 12 months | 0.00 [0.00, 1.00] | 0.00 [0.00, 1.00] | 0.262 |
| ICS | 468 (76.1%) | 18 (62%) | 0.135 |
| ICS dosage |  |  |  |
| LAMA | 172 (28%) | 4 (13.8%) | 0.144 |
| LTRA | 94 (15.3%) | 4 (13.8%) | 1.000 |
| Theophylline | 9 (1.5%) | 5 (17.2%) | **<0.001** |
| OCS maintenance | 152 (24.7%) | 7 (24.1%) | 1.000 |
| ICS category |  |  | 0.699 |
| 0-200 mg BED | 152 (24.7%) | 10 (34.5%) |  |
| 201-500 mg BED | 51 (8.3%) | 2 (6.9%) |  |
| 501-1000 mg BED | 114 (18.5%) | 5 (17.2%) |  |
| >1000mg BED | 298 (48.5%) | 12 (41.4%) |  |
| Biological |  |  | 0.785 |
| No biological | 552 (89.8%) | 28 (96.5%) |  |
| Mepolizumab | 34 (5.5%) | 1 (3.4%)om |  |
| Omalizumab | 22 (3.6%) | 0 (0.0%) |  |
| Reslizumab | 6 (1.0%) | 0 (0.0%) |  |
| Dupilumab | 1 (0.2%) | 0 (0.0%) |  |
| Patient reported triggers | | | |
| Flu like symptoms | 324 (52.68) | 15 (51.72) | 1.000 |
| Allergen exposure | 50 (8.13) | 1 (3.45) | 0.575 |
| Non-specific trigger | 54 (8.78) | 7 (24.14) | **0.015** |
| Infiltrate on X-ray or CT-scan N=576 | 60 (10.97) | 5 (17.24) | 0.460 |
| Virus infection | | | |
| Any virus N=135 | 74 (61.7%) | 10 (66.7%) | 0.925 |
| Rhinovirus N=135 | 24 (20.0%) | 9 (60.0%) | **0.002** |
| Influenza N=135 | 29 (24.2%) | 0 (0.0%) | 0.058 |
| HMPV N=135 | 8 (6.7%) | 0 (0.0%) | 0.652 |
| RSV N=135 | 6 (5.0%) | 0 (0.0%) | 0.825 |
| Parainfluenza N=135 | 4 (3.3%) | 0 (0.0%) | 1.000 |
| Corona N=135 | 8 (6.7%) | 1 (6.7%) | 1.000 |
| Enterovirus N=135 | 1 (0.8%) | 1 (6.7%) | 0.529 |
| Bacterial infection | | | |
| Any bacteria N=140 | 21 (16.8%) | 2 (12.5%) | 0.937 |
| Streptococcus Pneumoniae N=140 | 4 (3.2%) | 0 (0.0%) | 1.000 |
| Moraxella Catarrhalis N=140 | 1 (0.8%) | 1 (6.2%) | 0.543 |
| Hemophilus Influenzae N=140 | 14 (11.3%) | 1 (6.2%) | 0.854 |
| Staphylococcus Aureus N=140 | 1 (0.8%) | 1 (6.2%) | 0.543 |
| Aspergillus Fumigatus N=140 | 6 (4.9%) | 0 (0.0%) | 0.803 |
| Klebsiella Oxytoca N=140 | 2 (1.6%) | 0 (0.0%) | 1.000 |
| Pseudomonas aeruginosa N=140 | 2 (1.6%) | 0 (0.0%) | 1.000 |
| Arterial blood gas and oxygenation | | | |
| pH N= 379 | 7.45 [7.43, 7.49] | 7.41 [7.36, 7.47] | **0.001** |
| PaCO2 (kPa) N= 379 | 4.60 [4.10, 5.00] | 5.10 [4.40, 6.00] | **0.007** |
| PaO2 (kPa) N= 379 | 10.40 [9.00, 12.70] | 10.90 [8.20, 18.90] | 0.342 |
| Hypercapnia N=379 | 8 (2.3%) | 7 (24.1%) | **<0.001** |
| O2 (%) N= 379 | 94.70 [92.50, 96.30] | 95.65 [89.22, 97.72] | 0.445 |
| FiO2 (%) | 20.00 [20.00, 20.00] | 20.00 [20.00, 32.00] | **<0.001** |
| SpO2 (pulse oximetry) N=638 | 96.00 [93.00, 98.00] | 92.00 [89.00, 97.00] | **0.001** |
| Clinical outcome and disease severity | | | |
| NEWS N=622 | 3.00 [1.00, 5.00] | 7.00 [4.75, 8.25] | **<0.001** |
| Length of stay N=611 | 0.00 [0.00, 2.00] | 7.00 [3.00, 9.00] | **<0.001** |
| SpO2/FiO2 | 462.97 ± 51.12 | 360.50 ± 132.18 | **<0.001** |

The total number of exacerbations used to describe proportions was N=644, unless stated otherwise. Data is presented as proportion (percentage), mean ± SD or median [IQR]. BED = Beclomethasone Equivalent Dose, BMI = Body Mass Index, COPD = Chronic Obstructive Pulmonary Disease, CRP = c-reactive protein, CRSsNP = Chronic rhinosinusitis without nasal polyps, CRSwNP = Chronic rhinosinusitis with nasal polyps, CT = Computed Tomography, FEV1 = Forced Expiratory Volume in 1 second, FiO2 = Fraction of inspired oxygen , FVC= Forced Vital Capacity, GERD = Gastroesophageal Reflux Disease, HMPV = Human Metapneumovirus, ICS = Inhalation Corticosteroid, ICU = Intensive Care Unit, LAMA = Long Acting Muscarinic Antagonist, LTRA = Leukotriene Receptor Antagonists, NEWS = National Early Warning Score, OCS = Oral Corticosteroids, OSAS = Obstructive Sleep Apnea Syndrome, PaCO2= partial pressure of carbon dioxide, PaO2= partial pressure of oxygen, PEF = Peak Expiratory Flow, RSV = Respiratory Syncytial Virus, SpO2 = Saturation of Peripheral Oxygen

# eTable 8: Complete table of associations between study characteristics and disease severity and clinical outcomes

|  | Admission | | ICU admission | | Length of hospital stay | | SpO2/FiO2 | | NEWS | |
| --- | --- | --- | --- | --- | --- | --- | --- | --- | --- | --- |
| Parameter | Estimate (95% CI) | Adjusted p-value | Estimate (95% CI) | Adjusted p-value | Estimate (95% CI) | Adjusted p-value | Estimate (95% CI) | Adjusted p-value | Estimate (95% CI) | Adjusted p-value |
| Patient characteristics | | | | | | | | | | |
| Age | 0.002 (0.001) | 0.369 | -0.001 (0.001) | 0.580 | 0.02 (0.01) | 0.137 | -0.18 (0.17) | 0.830 | 0.01 (0.01) | 0.305 |
| Smoking status | NA | 0.769 | NA | 0.950 | NA | 0.351 | NA | 0.019 | NA | 0.292 |
| Past smoker | 0.069 (0.054) | NA | 0.008 (0.023) | NA | 0.59 (0.38) | NA | -12.14 (7.05) | NA | 0.51 (0.28) | NA |
| Current smoker | 0.017 (0.058) | NA | 0.009 (0.024) | NA | -0.36 (0.41) | NA | -27.36 (7.45) | NA | 0.54 (0.29) | NA |
| BMI (kg/m^2^) | 0.004 (0.004) | 0.690 | 0.003 (0.002) | 0.580 | 0.09 (0.03) | 0.022 | 0.10 (0.52) | 0.987 | 0.02 (0.02) | 0.670 |
| Ethnicity | NA | 0.862 | NA | 0.817 | NA | 0.801 | NA | 0.987 | NA | 0.739 |
| Black | -0.070 (0.067) | NA | -0.007 (0.029) | NA | -0.44 (0.46) | NA | -7.26 (9.12) | NA | 0.39 (0.35) | NA |
| Asian or pacific islander | -0.117 (0.100) | NA | -0.033 (0.043) | NA | -0.89 (0.67) | NA | 3.64 (13.23) | NA | 0.38 (0.51) | NA |
| Hispanic | -0.164 (0.164) | NA | -0.052 (0.069) | NA | -1.33 (1.07) | NA | -22.49 (21.12) | NA | -0.83 (0.82) | NA |
| Mixed | -0.010 (0.092) | NA | 0.065 (0.039) | NA | -0.24 (0.62) | NA | -6.55 (12.17) | NA | -0.43 (0.47) | NA |
| Other | -0.057 (0.072) | NA | -0.028 (0.031) | NA | -0.29 (0.48) | NA | 2.53 (9.75) | NA | -0.02 (0.38) | NA |
| Sex (female) | 0.059 (0.046) | 0.588 | -0.002 (0.020) | 0.950 | 0.40 (0.33) | 0.548 | 19.05 (5.98) | 0.020 | -0.21 (0.24) | 0.670 |
| Allergic sensitization to aeroallergens | -0.005 (0.048) | 0.958 | -0.018 (0.020) | 0.817 | -0.78 (0.34) | 0.137 | 6.03 (6.37) | 0.917 | -0.25 (0.25) | 0.638 |
| Allergic sensitization to Aspergillus Fumigatus | -0.140 (0.077) | 0.329 | -0.067 (0.043) | 0.580 | -0.77 (0.62) | 0.548 | 2.10 (10.02) | 0.987 | 0.28 (0.41) | 0.720 |
| Comorbidities | | | | | | | | | | |
| Comorbid chronic or allergic rhinitis | 0.024 (0.046) | 0.827 | 0.005 (0.020) | 0.930 | -0.24 (0.33) | 0.753 | 2.75 (6.18) | 0.987 | -0.28 (0.24) | 0.559 |
| Comorbid bronchiectasis | -0.007 (0.087) | 0.966 | -0.051 (0.037) | 0.640 | 0.39 (0.63) | 0.801 | -8.26 (12.17) | 0.987 | -0.10 (0.47) | 0.957 |
| Comorbid chronic obstructive pulmonary disease | 0.065 (0.061) | 0.639 | -0.011 (0.026) | 0.853 | 0.75 (0.45) | 0.351 | -5.20 (8.23) | 0.987 | 0.59 (0.32) | 0.240 |
| Comorbid chronic rhinosinusitis without nasal polyps | 0.038 (0.073) | 0.827 | 0.020 (0.031) | 0.819 | 0.20 (0.52) | 0.827 | 2.42 (9.90) | 0.987 | -0.07 (0.38) | 0.957 |
| Comorbid chronic rhinosinusitis with nasal polyps | 0.065 (0.070) | 0.702 | -0.022 (0.030) | 0.819 | 0.41 (0.51) | 0.727 | 10.20 (9.44) | 0.830 | -0.01 (0.37) | 0.968 |
| Comorbid Gastroesophageal reflux disease | 0.037 (0.059) | 0.827 | -0.006 (0.025) | 0.935 | 0.69 (0.43) | 0.351 | 17.18 (7.88) | 0.188 | -0.45 (0.31) | 0.418 |
| Comorbid urticaria | -0.047 (0.097) | 0.827 | -0.030 (0.042) | 0.819 | -0.29 (0.70) | 0.827 | 1.00 (13.12) | 0.987 | -0.44 (0.51) | 0.670 |
| Exacerbation characteristics | | | | | | | | | | |
| Peak flow % of predicted | -0.004 (0.002) | 0.216 | -0.001 (0.001) | 0.756 | -0.01 (0.01) | 0.591 | 0.31 (0.23) | 0.614 | -0.03 (0.01) | 0.001 |
| Season | NA | 0.864 | NA | 0.137 | NA | 0.827 | NA | 0.987 | NA | 0.957 |
| Spring | 0.053 (0.057) | NA | -0.072 (0.023) | NA | -0.07 (0.38) | NA | 2.26 (6.74) | NA | -0.06 (0.27) | NA |
| Summer | 0.017 (0.056) | NA | -0.055 (0.023) | NA | -0.39 (0.37) | NA | 9.04 (6.72) | NA | -0.08 (0.27) | NA |
| Winter | 0.051 (0.053) | NA | -0.051 (0.022) | NA | -0.26 (0.35) | NA | 2.65 (6.31) | NA | 0.12 (0.26) | NA |
| Duration of symptoms (days) | 0.000 (0.001) | 0.910 | 0.000 (0.000) | 0.620 | 0.00 (0.00) | 0.727 | 0.13 (0.08) | 0.376 | -0.01 (0.00) | 0.052 |
| Flu like symptoms | 0.022 (0.039) | 0.827 | -0.002 (0.016) | 0.950 | 0.13 (0.26) | 0.801 | -2.11 (4.70) | 0.987 | 0.42 (0.19) | 0.134 |
| Non-specific trigger exposure | -0.003 (0.067) | 0.969 | 0.067 (0.028) | 0.137 | -0.19 (0.44) | 0.827 | 13.07 (8.02) | 0.403 | -0.45 (0.32) | 0.451 |
| Allergen exposure | -0.077 (0.072) | 0.639 | -0.020 (0.030) | 0.819 | -0.79 (0.49) | 0.351 | 1.88 (8.78) | 0.987 | -0.55 (0.35) | 0.346 |
| Infiltrate on X-ray or CT-scan | 0.235 (0.066) | 0.024 | 0.030 (0.029) | 0.756 | 1.51 (0.45) | 0.022 | -17.31 (8.38) | 0.212 | 1.50 (0.32) | 0.000 |
| Lung function | | | | | | | | | | |
| FEV1 % of predicted | -0.003 (0.001) | 0.183 | -0.001 (0.001) | 0.756 | -0.02 (0.01) | 0.111 | 0.55 (0.15) | 0.007 | -0.02 (0.01) | 0.003 |
| FVC % of predicted | 0.000 (0.001) | 0.910 | 0.000 (0.001) | 0.819 | -0.01 (0.01) | 0.351 | 0.31 (0.17) | 0.307 | -0.02 (0.01) | 0.076 |
| FEV1/FVC | -0.503 (0.181) | 0.086 | -0.069 (0.083) | 0.819 | -2.20 (1.28) | 0.351 | 103.50 (23.47) | 0.001 | -3.63 (0.93) | 0.001 |
| Use of maintenance medication | | | | | | | | | | |
| Using inhalation corticosteroids | 0.026 (0.047) | 0.827 | -0.033 (0.020) | 0.558 | 0.58 (0.33) | 0.351 | 5.57 (5.99) | 0.917 | -0.16 (0.24) | 0.720 |
| ICS dosage in mcg BED x 1000 | 0.000 (0.000) | 0.933 | 0.000 (0.000) | 0.756 | 0.00 (0.00) | 0.866 | 0.01 (0.00) | 0.022 | 0.00 (0.00) | 0.305 |
| Using theophylline | 0.220 (0.143) | 0.460 | 0.275 (0.059) | 0.000 | 1.05 (0.99) | 0.637 | -41.46 (18.88) | 0.188 | 2.76 (0.74) | 0.002 |
| Using inhaled LAMA | 0.124 (0.050) | 0.138 | -0.027 (0.022) | 0.714 | 1.07 (0.35) | 0.040 | 2.07 (6.54) | 0.987 | 0.48 (0.26) | 0.240 |
| Using LTRA | -0.004 (0.061) | 0.967 | -0.011 (0.026) | 0.853 | -0.08 (0.43) | 0.911 | 11.36 (7.88) | 0.549 | -0.82 (0.31) | 0.052 |
| Using OCS | 0.142 (0.051) | 0.086 | 0.007 (0.022) | 0.906 | 0.71 (0.36) | 0.251 | 0.24 (6.64) | 0.987 | 0.19 (0.26) | 0.720 |
| Using a biological | -0.075 (0.070) | 0.639 | -0.041 (0.029) | 0.620 | -0.25 (0.47) | 0.801 | 0.35 (8.66) | 0.987 | 0.06 (0.35) | 0.957 |
| Using Mepolizumab | 0.006 (0.089) | NA | -0.025 (0.037) | NA | 0.47 (0.61) | NA | -12.46 (10.94) | NA | 0.52 (0.44) | NA |
| Using Omalizumab | -0.247 (0.114) | NA | -0.072 (0.048) | NA | -1.59 (0.79) | NA | 18.24 (14.27) | NA | -0.74 (0.57) | NA |
| Using Reslizumab | 0.156 (0.235) | NA | -0.039 (0.099) | NA | 0.20 (1.59) | NA | 18.84 (28.96) | NA | -0.73 (1.16) | NA |
| Using Dupilumab | -0.342 (0.488) | NA | -0.038 (0.203) | NA | -1.23 (3.21) | NA | 27.92 (58.19) | NA | 2.51 (2.35) | NA |
| Blood values | | | | | | | | | | |
| Leukocyte blood count | 0.010 (0.005) | 0.276 | 0.009 (0.002) | 0.002 | 0.06 (0.04) | 0.384 | -1.71 (0.66) | 0.078 | 0.12 (0.03) | 0.000 |
| Eosinophil blood count | 0.035 (0.059) | 0.827 | 0.003 (0.026) | 0.950 | -0.02 (0.42) | 0.973 | -6.44 (7.71) | 0.987 | -0.28 (0.30) | 0.670 |
| Neutrophil blood count | 0.013 (0.006) | 0.216 | 0.013 (0.003) | 0.000 | 0.09 (0.04) | 0.182 | -2.63 (0.75) | 0.009 | 0.15 (0.03) | 0.000 |
| CRP | 0.002 (0.001) | 0.030 | 0.000 (0.000) | 0.819 | 0.00 (0.00) | 0.717 | -0.03 (0.08) | 0.987 | 0.01 (0.00) | 0.001 |
| Infections | | | | | | | | | | |
| Any viral infection | 0.019 (0.086) | 0.910 | 0.021 (0.054) | 0.855 | -0.24 (0.83) | 0.866 | -5.03 (11.84) | 0.987 | 0.28 (0.49) | 0.752 |
| Rhinovirus infection | 0.079 (0.097) | 0.769 | 0.191 (0.060) | 0.023 | 0.11 (0.96) | 0.951 | -6.80 (13.28) | 0.987 | 0.44 (0.55) | 0.720 |
| Influenza infection | -0.021 (0.101) | 0.910 | -0.147 (0.067) | 0.230 | -1.22 (0.99) | 0.548 | 4.33 (14.62) | 0.987 | 0.15 (0.58) | 0.957 |
| HMPV infection | 0.120 (0.176) | 0.827 | -0.098 (0.110) | 0.817 | 1.21 (1.68) | 0.753 | -2.08 (24.08) | 0.987 | 0.04 (1.00) | 0.968 |
| RSV infection | 0.043 (0.202) | 0.910 | -0.063 (0.115) | 0.819 | 1.09 (1.82) | 0.801 | -14.68 (23.24) | 0.987 | -0.80 (1.12) | 0.720 |
| Parainfluenza infection | -0.656 (0.239) | 0.086 | -0.106 (0.157) | 0.819 | -3.30 (2.36) | 0.486 | 23.49 (35.66) | 0.987 | -2.74 (1.38) | 0.223 |
| Coronavirus infection | -0.087 (0.167) | 0.827 | 0.011 (0.098) | 0.950 | 1.36 (1.55) | 0.727 | 9.63 (20.80) | 0.987 | -0.11 (0.93) | 0.965 |
| Enterovirus infection | 0.368 (0.344) | 0.639 | 0.645 (0.185) | 0.015 | 2.04 (3.26) | 0.801 | 15.02 (45.32) | 0.987 | -2.56 (1.94) | 0.466 |
| Any bacterial infection | -0.061 (0.108) | 0.827 | -0.036 (0.073) | 0.819 | 0.99 (0.96) | 0.650 | 3.11 (17.63) | 0.987 | -0.69 (0.61) | 0.570 |
| Streptococcus spp infection | -0.297 (0.232) | 0.588 | -0.114 (0.162) | 0.819 | -1.57 (2.10) | 0.753 | 29.62 (37.88) | 0.987 | -0.57 (1.33) | 0.847 |
| Moraxella spp infection | -0.152 (0.324) | 0.827 | 0.391 (0.226) | 0.543 | -0.04 (2.94) | 0.988 | -138.80 (53.05) | 0.078 | 0.71 (1.87) | 0.875 |
| Hemophilus spp infection | 0.044 (0.131) | 0.883 | -0.052 (0.088) | 0.819 | 1.01 (1.17) | 0.727 | -0.07 (21.14) | 0.998 | -0.49 (0.74) | 0.720 |
| Staphylococcus spp infection | -0.133 (0.347) | 0.864 | 0.389 (0.226) | 0.543 | 1.58 (3.04) | 0.801 | -31.21 (55.24) | 0.987 | 0.31 (1.93) | 0.957 |
| Aspergillus spp infection | -0.381 (0.189) | 0.246 | -0.119 (0.134) | 0.817 | -1.44 (1.74) | 0.727 | 1.63 (31.54) | 0.987 | -0.66 (1.10) | 0.739 |
| Klebsiella spp infection | 0.299 (0.332) | 0.715 | -0.114 (0.228) | 0.819 | 3.50 (2.97) | 0.574 | -87.75 (53.58) | 0.403 | 0.11 (1.89) | 0.968 |
| Pseudomonas spp infection | 0.359 (0.319) | 0.639 | -0.108 (0.227) | 0.819 | 9.46 (2.81) | 0.022 | -25.40 (52.97) | 0.987 | -0.28 (1.85) | 0.957 |
| Healthcare utilizations | | | | | | | | | | |
| Number of hospital admissions 12 months prior | 0.032 (0.023) | 0.558 | -0.010 (0.010) | 0.756 | 0.04 (0.15) | 0.866 | 2.59 (2.80) | 0.917 | -0.12 (0.11) | 0.638 |
| Emergency department visits 12 months prior | 0.020 (0.015) | 0.588 | -0.007 (0.006) | 0.756 | -0.04 (0.10) | 0.827 | -0.15 (1.89) | 0.987 | -0.07 (0.08) | 0.670 |
| Number of asthma exacerbations 12 months prior | 0.016 (0.014) | 0.639 | -0.006 (0.006) | 0.756 | -0.04 (0.10) | 0.827 | 0.34 (1.76) | 0.987 | -0.09 (0.07) | 0.526 |
| Courses systemic corticosteroids in the 12 months prior | 0.022 (0.013) | 0.369 | -0.005 (0.005) | 0.819 | 0.01 (0.09) | 0.967 | -1.05 (1.61) | 0.987 | 0.00 (0.07) | 0.968 |
| Time since last emergency presentation | 0.000 (0.000) | 0.769 | 0.000 (0.000) | 0.950 | 0.00 (0.00) | 0.866 | -0.01 (0.03) | 0.987 | 0.00 (0.00) | 0.755 |
| Time since last hospital admission | -0.001 (0.000) | 0.216 | 0.000 (0.000) | 0.979 | 0.00 (0.00) | 0.697 | 0.01 (0.04) | 0.987 | 0.00 (0.00) | 0.720 |
| Time since last exacerbation | 0.000 (0.000) | 0.827 | 0.000 (0.000) | 0.950 | 0.00 (0.00) | 0.801 | 0.02 (0.04) | 0.987 | 0.00 (0.00) | 0.720 |

BED = Beclomethasone Equivalent Dose, BMI = Body Mass Index , CRP = c-reactive protein, CT = Computed Tomography, FiO2 = Fraction of inspired oxygen, FEV1 = Forced Expiratory Volume in 1 second, FVC = Forced Vital Capacity, HMPV = Human Metapneumovirus, ICS = Inhalation Corticosteroid, ICU = Intensive Care Unit, LAMA = Long-Acting Muscarinic Antagonist, LTRA = Leukotriene Receptor Antagonists, NEWS = National Early Warning Score, OCS = Oral Corticosteroid, RSV = Respiratory Syncytial Virus, SpO2= Saturation of Peripheral Oxygen

# eTable 9: Variables selected for the prediction of hospital admission

| Variable | Sum Sq | Mean Sq | NumDF | DenDF | F value | P value |
| --- | --- | --- | --- | --- | --- | --- |
| Infiltrate on X-ray or CT-scan | 2,982 | 1,491 | 2 | 597,6 | 7,744 | 0,000 |
| C-reactive protein (CRP) | 1,226 | 1,226 | 1 | 595,1 | 6,367 | 0,012 |
| Using oral corticosteroids | 0,930 | 0,930 | 1 | 549,2 | 4,832 | 0,028 |
| Eosinophil blood count | 0,615 | 0,615 | 1 | 510,2 | 3,194 | 0,075 |
| Sex | 0,597 | 0,597 | 1 | 291,3 | 3,101 | 0,079 |
| Using a biological | 0,490 | 0,490 | 1 | 563,8 | 2,546 | 0,111 |
| Dosage of inhalation corticosteroids | 0,365 | 0,365 | 1 | 515,9 | 1,898 | 0,169 |
| Using inhaled long-acting muscarinic antagonists | 0,302 | 0,302 | 1 | 406,0 | 1,566 | 0,211 |
| Using leukotriene receptor antagonists | 0,280 | 0,280 | 1 | 329,1 | 1,455 | 0,229 |
| Using theophylline | 0,215 | 0,215 | 1 | 386,9 | 1,117 | 0,291 |
| Allergen exposure | 0,185 | 0,185 | 1 | 603,6 | 0,963 | 0,327 |
| Age | 0,168 | 0,168 | 1 | 307,9 | 0,874 | 0,351 |
| Allergic sensitization to aeroallergens | 0,380 | 0,190 | 2 | 323,1 | 0,987 | 0,374 |
| Neutrophil blood count | 0,134 | 0,134 | 1 | 606,9 | 0,694 | 0,405 |
| Categorical inhalation corticosteroid dosage | 0,453 | 0,151 | 3 | 566,8 | 0,784 | 0,503 |
| Total number of emergency department visits | 0,076 | 0,076 | 1 | 51,5 | 0,397 | 0,532 |
| Number of asthma exacerbations 12 months prior | 0,063 | 0,063 | 1 | 480,0 | 0,328 | 0,567 |
| Emergency department visits 12 months prior | 0,057 | 0,057 | 1 | 601,7 | 0,298 | 0,586 |
| Ethnicity | 0,829 | 0,138 | 6 | 349,6 | 0,718 | 0,636 |
| Courses systemic corticosteroids in the 12 months prior | 0,039 | 0,039 | 1 | 604,2 | 0,203 | 0,653 |
| Using inhalation corticosteroids | 0,027 | 0,027 | 1 | 566,5 | 0,141 | 0,707 |
| Comorbid chronic rhinosinusitis with nasal polyps | 0,024 | 0,024 | 1 | 190,1 | 0,126 | 0,723 |
| Number of hospital admissions 12 months prior | 0,009 | 0,009 | 1 | 602,2 | 0,049 | 0,825 |
| Smoking status | 0,132 | 0,044 | 3 | 389,955 | 0,228 | 0,877 |
| Leukocyte blood count | 0,003 | 0,003 | 1 | 600,470 | 0,014 | 0,905 |

# eTable 10: Variables selected for the prediction of intensive care unit admission

| Variable | Sum Sq | Mean Sq | NumDF | DenDF | F value | P value |
| --- | --- | --- | --- | --- | --- | --- |
| Using theophylline | 0,653 | 0,653 | 1 | 396,87 | 19,705 | 0,000 |
| Neutrophil blood count | 0,341 | 0,341 | 1 | 595,82 | 10,297 | 0,001 |
| Season | 0,336 | 0,112 | 3 | 586,32 | 3,383 | 0,018 |
| Non-specific trigger exposure | 0,174 | 0,174 | 1 | 593,91 | 5,257 | 0,022 |
| Infiltrate on X-ray or CT-scan | 0,199 | 0,100 | 2 | 584,08 | 3,006 | 0,050 |
| Using inhalation corticosteroids | 0,073 | 0,073 | 1 | 561,27 | 2,190 | 0,139 |
| Duration of symptoms | 0,062 | 0,062 | 1 | 580,42 | 1,857 | 0,173 |
| Age | 0,045 | 0,045 | 1 | 314,06 | 1,371 | 0,243 |
| Allergen exposure | 0,043 | 0,043 | 1 | 592,20 | 1,295 | 0,256 |
| Using inhaled long-acting muscarinic antagonists | 0,042 | 0,042 | 1 | 397,58 | 1,273 | 0,260 |
| C-reactive protein (CRP) | 0,042 | 0,042 | 1 | 586,54 | 1,259 | 0,262 |
| Dosage of inhalation corticosteroids | 0,037 | 0,037 | 1 | 505,20 | 1,104 | 0,294 |
| Using a biological | 0,036 | 0,036 | 1 | 547,10 | 1,074 | 0,300 |
| Comorbid chronic rhinosinusitis with nasal polyps | 0,036 | 0,036 | 1 | 211,48 | 1,075 | 0,301 |
| Comorbid bronchiectasis | 0,032 | 0,032 | 1 | 186,03 | 0,964 | 0,327 |
| Leukocyte blood count | 0,030 | 0,030 | 1 | 586,94 | 0,919 | 0,338 |
| Using oral corticosteroids | 0,024 | 0,024 | 1 | 538,69 | 0,737 | 0,391 |
| Allergic sensitization to aeroallergens | 0,054 | 0,027 | 2 | 352,81 | 0,813 | 0,444 |
| Comorbid chronic or allergic rhinitis | 0,018 | 0,018 | 1 | 292,28 | 0,551 | 0,459 |
| Comorbid urticaria | 0,013 | 0,013 | 1 | 224,64 | 0,402 | 0,527 |
| Flu like symptoms | 0,013 | 0,013 | 1 | 596,00 | 0,392 | 0,532 |
| Courses systemic corticosteroids in the 12 months prior | 0,012 | 0,012 | 1 | 595,93 | 0,370 | 0,544 |
| Eosinophil blood count | 0,011 | 0,011 | 1 | 515,05 | 0,338 | 0,561 |
| Ethnicity | 0,161 | 0,027 | 6 | 351,68 | 0,809 | 0,563 |
| Categorical inhalation corticosteroid dosage | 0,067 | 0,022 | 3 | 557,30 | 0,670 | 0,571 |
| Using leukotriene receptor antagonists | 0,010 | 0,010 | 1 | 325,43 | 0,289 | 0,591 |
| Smoking status | 0,050 | 0,017 | 3 | 397,75 | 0,507 | 0,678 |
| Comorbid chronic obstructive pulmonary disease | 0,005 | 0,005 | 1 | 226,57 | 0,157 | 0,692 |
| Comorbid chronic rhinosinusitis without nasal polyps | 0,002 | 0,002 | 1 | 219,86 | 0,067 | 0,796 |
| Number of asthma exacerbations 12 months prior | 0,002 | 0,002 | 1 | 479,94 | 0,062 | 0,804 |
| Comorbid obstructive sleep apnea syndrome | 0,001 | 0,001 | 1 | 245,95 | 0,044 | 0,834 |
| Emergency department visits 12 months prior | 0,000 | 0,000 | 1 | 537,12 | 0,009 | 0,924 |
| Sex | 0,000 | 0,000 | 1 | 316,29 | 0,001 | 0,973 |
| Total number of emergency department visits | 0,000 | 0,000 | 1 | 51,02 | 0,000 | 0,990 |

# eTable 11: Variables selected for the prediction of National Early Warning Score (NEWS)

| Variable | Sum Sq | Mean Sq | NumDF | DenDF | F value | P value |
| --- | --- | --- | --- | --- | --- | --- |
| Using theophylline | 67,916 | 67,916 | 1 | 383,728 | 16,663 | 0,000 |
| Using leukotriene receptor antagonists | 51,569 | 51,569 | 1 | 328,305 | 12,652 | 0,000 |
| Infiltrate on X-ray or CT-scan | 63,434 | 31,717 | 2 | 585,945 | 7,782 | 0,000 |
| Duration of symptoms | 38,924 | 38,924 | 1 | 585,578 | 9,550 | 0,002 |
| Using oral corticosteroids | 11,711 | 11,711 | 1 | 546,961 | 2,873 | 0,091 |
| Number of asthma exacerbations 12 months prior | 9,743 | 9,743 | 1 | 467,899 | 2,390 | 0,123 |
| C-reactive protein (CRP) | 7,376 | 7,376 | 1 | 589,343 | 1,810 | 0,179 |
| Courses systemic corticosteroids in the 12 months prior | 6,943 | 6,943 | 1 | 592,997 | 1,704 | 0,192 |
| Allergen exposure | 6,938 | 6,938 | 1 | 592,179 | 1,702 | 0,193 |
| Using inhaled long-acting muscarinic antagonists | 6,609 | 6,609 | 1 | 436,676 | 1,621 | 0,204 |
| Dosage of inhalation corticosteroids | 5,726 | 5,726 | 1 | 554,856 | 1,405 | 0,236 |
| Non-specific trigger exposure | 5,696 | 5,696 | 1 | 592,911 | 1,398 | 0,238 |
| Age | 5,325 | 5,325 | 1 | 295,169 | 1,307 | 0,254 |
| Allergic sensitization to aeroallergens | 10,559 | 5,279 | 2 | 326,884 | 1,295 | 0,275 |
| Neutrophil blood count | 4,448 | 4,448 | 1 | 590,755 | 1,091 | 0,297 |
| Comorbid gastroesophageal reflux disease | 4,077 | 4,077 | 1 | 206,018 | 1,000 | 0,318 |
| Comorbid bronchiectasis | 3,990 | 3,990 | 1 | 176,833 | 0,979 | 0,324 |
| Smoking status | 13,392 | 4,464 | 3 | 381,413 | 1,095 | 0,351 |
| Using inhalation corticosteroids | 3,215 | 3,215 | 1 | 552,259 | 0,789 | 0,375 |
| Leukocyte blood count | 3,040 | 3,040 | 1 | 591,578 | 0,746 | 0,388 |
| Comorbid urticaria | 2,583 | 2,583 | 1 | 214,043 | 0,634 | 0,427 |
| Ethnicity | 23,093 | 3,849 | 6 | 339,378 | 0,944 | 0,463 |
| Comorbid chronic obstructive pulmonary disease | 1,459 | 1,459 | 1 | 232,725 | 0,358 | 0,550 |
| Emergency department visits 12 months prior | 1,445 | 1,445 | 1 | 591,623 | 0,355 | 0,552 |
| Eosinophil blood count | 1,241 | 1,241 | 1 | 519,817 | 0,305 | 0,581 |
| Comorbid chronic or allergic rhinitis | 1,206 | 1,206 | 1 | 278,378 | 0,296 | 0,587 |
| Flu like symptoms | 1,064 | 1,064 | 1 | 588,936 | 0,261 | 0,610 |
| Comorbid obstructive sleep apnea syndrome | 0,937 | 0,937 | 1 | 224,731 | 0,230 | 0,632 |
| Sex | 0,702 | 0,702 | 1 | 298,360 | 0,172 | 0,678 |
| Categorical inhalation corticosteroid dosage | 3,994 | 1,331 | 3 | 562,610 | 0,327 | 0,806 |
| Total number of emergency department visits | 0,066 | 0,066 | 1 | 59,588 | 0,016 | 0,899 |
| Number of hospital admissions 12 months prior | 0,057 | 0,057 | 1 | 558,730 | 0,014 | 0,906 |

# eTable 12: Variables selected for the prediction of the ratio of peripheral arterial oxygen saturation to the inspired fraction of oxygen (SpO2/FiO2)

| Variable | Sum Sq | Mean Sq | NumDF | DenDF | F value | P value |
| --- | --- | --- | --- | --- | --- | --- |
| Infiltrate on X-ray or CT-scan | 31238 | 15619 | 2 | 586,6 | 6,271 | 0,002 |
| Using theophylline | 19614 | 19614 | 1 | 419,6 | 7,875 | 0,005 |
| Neutrophil blood count | 11761 | 11761 | 1 | 573,1 | 4,722 | 0,030 |
| Eosinophil blood count | 11079 | 11079 | 1 | 510,8 | 4,449 | 0,035 |
| Smoking status | 20158 | 6719 | 3 | 356,6 | 2,698 | 0,046 |
| Comorbid gastroesophageal reflux disease | 8719 | 8719 | 1 | 194,0 | 3,501 | 0,063 |
| Sex | 8634 | 8634 | 1 | 280,2 | 3,467 | 0,064 |
| Duration of symptoms | 8544 | 8544 | 1 | 586,4 | 3,430 | 0,065 |
| Non-specific trigger exposure | 7163 | 7163 | 1 | 584,2 | 2,876 | 0,090 |
| Using leukotriene receptor antagonists | 4421 | 4421 | 1 | 343,7 | 1,775 | 0,184 |
| Allergic sensitization to aeroallergens | 8023 | 4011 | 2 | 296,3 | 1,611 | 0,202 |
| Age | 2834 | 2834 | 1 | 277,7 | 1,138 | 0,287 |
| Emergency department visits 12 months prior | 2779 | 2779 | 1 | 588,6 | 1,116 | 0,291 |
| Comorbid chronic or allergic rhinitis | 2761 | 2761 | 1 | 256,7 | 1,109 | 0,293 |
| Dosage of inhalation corticosteroids | 2383 | 2383 | 1 | 579,6 | 0,957 | 0,328 |
| Season | 8046 | 2682 | 3 | 548,6 | 1,077 | 0,358 |
| Comorbid obstructive sleep apnea syndrome | 2097 | 2097 | 1 | 209,3 | 0,842 | 0,360 |
| Comorbid chronic obstructive pulmonary disease | 1867 | 1867 | 1 | 221,2 | 0,749 | 0,388 |
| Comorbid chronic rhinosinusitis with nasal polyps | 1833 | 1833 | 1 | 190,5 | 0,736 | 0,392 |
| Number of hospital admissions 12 months prior | 1369 | 1369 | 1 | 476,1 | 0,550 | 0,459 |
| C-reactive protein (CRP) | 1346 | 1346 | 1 | 588,6 | 0,540 | 0,463 |
| Using oral corticosteroids | 1038 | 1038 | 1 | 573,2 | 0,417 | 0,519 |
| Categorical inhalation corticosteroid dosage | 5361 | 1787 | 3 | 567,0 | 0,717 | 0,542 |
| Leukocyte blood count | 809 | 809 | 1 | 587,6 | 0,325 | 0,569 |
| Comorbid urticaria | 699 | 699 | 1 | 195,9 | 0,281 | 0,597 |
| Using a biological | 666 | 666 | 1 | 585,5 | 0,267 | 0,605 |
| Number of asthma exacerbations 12 months prior | 641 | 641 | 1 | 460,3 | 0,257 | 0,612 |
| Flu like symptoms | 583 | 583 | 1 | 570,1 | 0,234 | 0,629 |
| Comorbid chronic rhinosinusitis without nasal polyps | 563 | 563 | 1 | 204,8 | 0,226 | 0,635 |
| Using inhalation corticosteroids | 522 | 522 | 1 | 539,4 | 0,209 | 0,647 |
| Comorbid bronchiectasis | 383 | 383 | 1 | 172,4 | 0,154 | 0,695 |
| Total number of emergency department visits | 225 | 225 | 1 | 63,3 | 0,090 | 0,765 |
| Allergen exposure | 205 | 205 | 1 | 588,6 | 0,082 | 0,774 |
| Ethnicity | 6298 | 1050 | 6 | 309,6 | 0,421 | 0,865 |
| Using inhaled long-acting muscarinic antagonists | 11 | 11 | 1 | 462,7 | 0,005 | 0,946 |

# eTable 13: Variables selected for the prediction of length of hospital stay

| Variable | Sum Sq | Mean Sq | NumDF | DenDF | F value | P value |
| --- | --- | --- | --- | --- | --- | --- |
| Infiltrate on X-ray or CT-scan | 61,157 | 30,579 | 2 | 578,6 | 3,886 | 0,021 |
| Dosage of inhalation corticosteroids | 33,821 | 33,821 | 1 | 568,6 | 4,298 | 0,039 |
| Using leukotriene receptor antagonists | 30,301 | 30,301 | 1 | 396,8 | 3,850 | 0,050 |
| Using inhaled long-acting muscarinic antagonists | 20,015 | 20,015 | 1 | 483,3 | 2,543 | 0,111 |
| Using oral corticosteroids | 17,929 | 17,929 | 1 | 572,0 | 2,278 | 0,132 |
| Neutrophil blood count | 17,132 | 17,132 | 1 | 568,1 | 2,177 | 0,141 |
| Sex | 13,077 | 13,077 | 1 | 344,3 | 1,662 | 0,198 |
| Comorbid gastroesophageal reflux disease | 10,158 | 10,158 | 1 | 254,0 | 1,291 | 0,257 |
| Eosinophil blood count | 9,128 | 9,128 | 1 | 523,7 | 1,160 | 0,282 |
| Using a biological | 6,753 | 6,753 | 1 | 579,7 | 0,858 | 0,355 |
| Comorbid obstructive sleep apnea syndrome | 5,887 | 5,887 | 1 | 275,2 | 0,748 | 0,388 |
| Allergen exposure | 5,789 | 5,789 | 1 | 579,7 | 0,736 | 0,391 |
| Duration of symptoms | 4,337 | 4,337 | 1 | 579,5 | 0,551 | 0,458 |
| Categorical inhalation corticosteroid dosage | 19,155 | 6,385 | 3 | 563,4 | 0,811 | 0,488 |
| Using theophylline | 3,308 | 3,308 | 1 | 488,6 | 0,420 | 0,517 |
| Allergic sensitization to aeroallergens | 10,369 | 5,184 | 2 | 354,6 | 0,659 | 0,518 |
| C-reactive protein (CRP) | 2,974 | 2,974 | 1 | 579,9 | 0,378 | 0,539 |
| Leukocyte blood count | 2,932 | 2,932 | 1 | 578,1 | 0,373 | 0,542 |
| Smoking status | 15,680 | 5,227 | 3 | 402,7 | 0,664 | 0,574 |
| Courses systemic corticosteroids in the 12 months prior | 2,375 | 2,375 | 1 | 576,0 | 0,302 | 0,583 |
| Total number of emergency department visits | 2,209 | 2,209 | 1 | 94,2 | 0,281 | 0,597 |
| Season | 13,290 | 4,430 | 3 | 551,6 | 0,563 | 0,640 |
| Flu like symptoms | 1,401 | 1,401 | 1 | 562,8 | 0,178 | 0,673 |
| Comorbid bronchiectasis | 1,058 | 1,058 | 1 | 229,0 | 0,134 | 0,714 |
| Non-specific trigger exposure | 0,994 | 0,994 | 1 | 576,3 | 0,126 | 0,722 |
| Age | 0,981 | 0,981 | 1 | 337,3 | 0,125 | 0,724 |
| Emergency department visits 12 months prior | 0,761 | 0,761 | 1 | 486,6 | 0,097 | 0,756 |
| Using inhalation corticosteroids | 0,699 | 0,699 | 1 | 544,0 | 0,089 | 0,766 |
| Comorbid chronic rhinosinusitis without nasal polyps | 0,432 | 0,432 | 1 | 263,4 | 0,055 | 0,815 |
| Comorbid chronic rhinosinusitis with nasal polyps | 0,268 | 0,268 | 1 | 245,6 | 0,034 | 0,854 |
| Ethnicity | 20,487 | 3,414 | 6 | 364,2 | 0,434 | 0,856 |
| Number of hospital admissions 12 months prior | 0,247 | 0,247 | 1 | 512,9 | 0,031 | 0,859 |
| Comorbid chronic obstructive pulmonary disease | 0,005 | 0,005 | 1 | 286,1 | 0,001 | 0,981 |
| Comorbid chronic or allergic rhinitis | 0,003 | 0,003 | 1 | 315,1 | 0,000 | 0,985 |
| Comorbid urticaria | 0,001 | 0,001 | 1 | 251,557 | 0,000 | 0,989 |

# eTable 14: P-values of variables selected in the LASSO models in a subgroup of patients without comorbid COPD

| Variable | hospital admission | ICU admission | NEWS | Oxygenation efficiency  (SpO2/FiO2) | Length of hospital stay |
| --- | --- | --- | --- | --- | --- |
| Infiltrate on X-ray or CT-scan | 0,000 | ns | 0,007 | 0,011 | 0,030 |
| Using oral corticosteroids | 0,004 | ns | ns | ns | ns |
| Comorbid obstructive sleep apnea syndrome | 0,032 | ns | ns | ns | ns |
| Using a biological | 0,035 | ns | ns | ns | ns |
| C-reactive protein (CRP) | 0,047 | ns | ns | ns | ns |
| Using theophylline | ns | 0,000 | 0,000 | 0,005 | ns |
| Neutrophil blood count | ns | 0,006 | ns | ns | ns |
| Season | ns | 0,008 | ns | ns | ns |
| Using leukotriene receptor antagonists | ns | ns | 0,005 | ns | ns |
| Leukocyte blood count | ns | ns | 0,044 | ns | ns |
| Smoking status | ns | ns | ns | 0,022 | ns |
| Eosinophil blood count | ns | ns | ns | 0,043 | ns |
| Dosage of inhalation corticosteroids | ns | ns | ns | ns | 0,020 |
| Using inhaled long-acting muscarinic antagonists | ns | ns | ns | ns | 0,031 |
| Comorbid gastroesophageal reflux disease | ns | ns | ns | ns | 0,043 |
